# Unravelling the gut bacteriome of *Ips* (Coleoptera: Curculionidae: Scolytinae): identifying core bacterial assemblage and their ecological relevance

**DOI:** 10.1038/s41598-020-75203-5

**Published:** 2020-10-29

**Authors:** Amrita Chakraborty, Muhammad Zubair Ashraf, Roman Modlinger, Jiří Synek, Fredrik Schlyter, Amit Roy

**Affiliations:** 1 EVA 4.0 Unit, Faculty of Forestry and Wood Sciences, Czech University of Life Sciences Prague, Kamýcká 129, Suchdol, 165 21 Prague 6, Czech Republic; 2 Excellent Team for Mitigation (ETM), Faculty of Forestry and Wood Sciences, Czech University of Life Sciences Prague, Kamýcká 129, Suchdol, 165 21 Prague 6, Czech Republic; 3 Department of Plant Protection Biology, Swedish University of Agricultural Sciences, 230 53 Alnarp, Sweden

**Keywords:** Microbial ecology, Microbial communities

## Abstract

Bark beetles often serve as forest damaging agents, causing landscape-level mortality. Understanding the biology and ecology of beetles are important for both, gathering knowledge about important forest insects and forest protection. Knowledge about the bark beetle gut-associated bacteria is one of the crucial yet surprisingly neglected areas of research with European tree-killing bark beetles. Hence, in this study, we survey the gut bacteriome from five *Ips* and one non-*Ips* bark beetles from Scolytinae. Results reveal 69 core bacterial genera among five *Ips* beetles that may perform conserved functions within the bark beetle holobiont. The most abundant bacterial genera from different bark beetle gut include *Erwinia, Sodalis, Serratia, Tyzzerella, Raoultella, Rahnella, Wolbachia, Spiroplasma, Vibrio,* and *Pseudoxanthomonas*. Notable differences in gut-associated bacterial community richness and diversity among the beetle species are observed. Furthermore, the impact of sampling location on the overall bark beetle gut bacterial community assemblage is also documented, which warrants further investigations. Nevertheless, our data expanded the current knowledge about core gut bacterial communities in *Ips* bark beetles and their putative function such as cellulose degradation, nitrogen fixation, detoxification of defensive plant compounds, and inhibition of pathogens, which could serve as a basis for further metatranscriptomics and metaproteomics investigations.

## Introduction

Apart from providing commodities such as food and wood, forests perform fundamental ecological services such as biodiversity preservation and climatic regulation^1^. Forest health in Europe is under severe threat due to global climatic change. The trade-off in tree carbon investment between primary and secondary metabolites is observed under abiotic stress conditions such as drought^2^. On top of impacting the tree defence physiology adversely, repeated years of drought also trigger more recurrent and devastating bark beetle outbreaks leading to large scale forest decline^3,4^. However, this is not true for healthy trees as conifers can produce entomotoxic defensive chemicals, such as monoterpenes, in addition to releasing anti-aggregation compound such as 4-allylanisole to defend themselves against bark beetle attacks^5–10^. Conifer defence primarily composed of terpenoid resins made up of mono-, sesqui, and diterpenes among which monoterpenes are already reported entomotoxic in higher concentrations to bark beetles^11,12^. Moreover, conifer tissue feeding is nutritionally limiting for bark beetles because of the low concentration of phosphorus, nitrogen, sterols, and vitamins^13^. Successful bark beetle colonization demands strategies to overcome the noxious effect of plant secondary metabolites such as avoidance of toxin ingestion by modification of feeding behaviour; plant defence manipulation by converting the highly toxic metabolites to low toxic ones; enhanced excretion of ingested toxins; sequestration of the toxin; target-site mutation or metabolic degradation of toxin compounds^14^. Using aggregation pheromone mediated mass attack, some bark beetles (European spruce bark beetle, *Ips typographus*) can surmount host defence as the conifers unable to accumulate sufficient defence response to beetles arrived *en masse*^15^. Non-aggressive bark beetles adapted to survive in the habitat with terpene using some alternative strategies such as having bacterial microsymbiont with higher tolerance to host terpenes, i.e. Red turpentine beetle (*Dendroctonus valens;* non-aggressive or occasionally aggressive) associated bacteria showed higher monoterpene tolerance compare to aggressive mountain pine beetle (*Dendroctonus ponderosae*)^16^.

During the last decade, the contribution of microbes in shaping up insect ecology came into the limelight^17–19^. Due to short generation time and enormous ability to adapt new environments made microbes an inevitable ally for most of the living organisms, including insects. Symbiotic microorganisms influence the insect-plant interaction by providing essential nutrients, degrading complex dietary polymers, and even plant secondary metabolites^20–22^. Gut microbes reported facilitating insect herbivory on toxin-laden host tissues^23^. However, gut microbes are often vulnerable to plant allelochemicals and served as a primary target to decrease insect herbivory^24,25^. Nevertheless, bacterial association with insects is widespread and more often adaptive.

Bark beetles co-exist in a hidden habitat with numerous bacterial strains within their gut, larvae, mycangia, oral secretions and galleries. The influence of microbes on bark beetle's life is unavoidable yet little known about them. Metabolic capabilities of bacteria can benefit bark beetles in several ways by providing certain nutrition (i.e. nitrogen) from the nutritionally limited host tissues^20,26,27^, inhibiting the antagonistic ones^28–30^, cellulose degradation^31–34^, pheromone production and detoxification of host toxin allelochemicals^35–40^. For instance, bacterial isolates from the coniferous subcortical habitat such as *Pseudomonas*, *Serratia,* and *Rahnella* that are also associated with *Dendroctonus valens* reported reducing and metabolizing the monoterpenes^35,36^. Similarly, *Erwinia typography* obtained from IT gut (*Ips typographus*) showed tolerance to high concentration of spruce monoterpene myrcene^41^. Recently, using the metagenomic approach, bacterial community harbouring genes associated with terpene degradation were identified from mountain pine beetles^16^. Unfortunately, there is very little information on whether terpene degradation via bacteria occurs inside the bark beetles or not. It is worth mentioning here that the potential of terpene degradation is not limited to bark beetle associated bacteria, i.e., some environmental bacteria such as *Burkholderia xenovorans*^42^ and *Pseudomonas abietaniphila* BKME-9^43^ can do that as well.

Despite the formidable defence of conifers, bark beetles can colonize and cause landscape-level mortality. It is not clear if the beetles manage the living in such a hostile, toxin-enriched environment on their own or in association with several associated microbes. Recent reports indicated that bark beetle holobiont (beetles together with its microsymbionts) faces the challenging host defence together^16,21, 35,36^. Unfortunately, information about the adaptive advantage due to the presence of the consortium of bacteria in the bark beetle gut is limited and confined in a few genera of beetles present in North America and China, i.e., mountain pine beetle, red turpentine beetles. There is also limited information about the gut-associated bacterial community of IT^44,45^ and other bark beetles from *Ips* species^46,47^, causing severe conifer mortality in Europe, although some reports are suggesting their crucial role in beetle survival^41^. Besides, microbiome studies on bark beetles that are performed on the whole body of the beetles often represent a mix of biota from the digestive tract, external biota that escapes surface sterilization (mostly from mouthparts and entomogenous fungi from exoskeleton), hemocoel and gonad rudiments. Hence, the current knowledge about the true association between bark beetles and their gut-associated bacteria is inadequate and demands further investigations. Furthermore, it will also be intriguing to know the core bacteriome in the gut of different bark beetle species (Coleoptera: Curculionidae: Scolytinae) and their ecological relevance, which may excavate surprising potentials for future bark beetle management. In the present study, we have screened the gut bacteriome of six economically important bark beetles from Scolytinae subfamily namely *Ips typographus* (European spruce bark beetle), *Ips duplicatus* (northern bark beetle), *Ips sexdentatus* (six-toothed bark beetle)*, Ips acuminatus* (pine engraver beetle), *Ips cembrae* (large larch bark beetle) and *Polygraphus poligraphus* (Small spruce bark beetle) collected from Czech forests using 16S amplicon sequencing.

*Ips typographus* (IT) is undoubtedly one of the most destructive pests of European spruce trees^48^. *Ips duplicatus* (ID) primarily attacks standing trees only, and often found on the same tree infested with IT^49^. Similarly, *Polygraphus poligraphus* (PP—non-*Ips* species) attacks spruce trees of middle age (41–60 years old), which are growing under dense and shaded condition. PP is also considered as a serious pest of spruce in some areas of Europe^49^. On the contrary, *Ips sexdentatus* (SX)^49^ is widely distributed as a secondary pest of pine with the capability of infesting other conifer species. SX prefers stressed, and weakened trees and often found together with *Ips acuminatus* (IAC), a species causing extensive damage at the top and on the branches of Scots pine, which is an important and widely distributed tree species in European forests^50^. *Ips cembrae* (IC) serves as a secondary pest of European larix population and like most other beetles in the study selects wind-blown and dying trees for colonization^49^. However, during an outbreak, IC can become a severe pest for not only larch but also for spruce. It is worth to mention here that all these beetles can attack green standing trees under drought conditions and thus possess an increasing threat to forests. In the last three decades, all these bark beetles contribute momentous damage to European forests. For instance, *Ips typographus* (L.) [IT] outbreak alone in Czechia was recorded volume 27,557,000 m^3^ of infested spruce wood from 2015^51,52^. Although available data from the whole of Europe are incomplete, at least 2,819,000 ha were attacked by IT between 1990 and 2001, resulting in the death of 31,643,000 m^3^ of spruce^48^. Therefore, in-depth knowledge about bark beetle adaptive ecology, including their gut symbiotic associations and core microbiome, is of utmost importance not only from the stand-point of enhancing biological understanding about important forest insects but also from the sustainable bark beetle management perspectives.

## Results

### Sequencing statistics

A total of 5,194,234 paired-end reads were generated after the sequencing of six different species of bark beetles (Coleoptera: Curculionidae: Scolytinae): *Ips typographus* (IT), *Ips duplicatus* (ID), *Ips cembrae* (IC); *Ips sexdentatus* (SX), *Ips acuminatus* (IAC) and *Polygraphus poligraphus* (PP). The quality control (QC) tests were performed based on quality score Q < 30 to be discarded, and around 4.5 million clean reads were obtained. The clean reads, found in each beetle species (IT-917,707; ID-884,296; IC-890,794; SX-898,824; IAC-494,122 and PP-737,615 reads), were then used for downstream bioinformatic processing (Supplementary excel 1, 2).

### Gut bacterial diversity in bark beetles

#### OTU abundance

**Figure 1:**
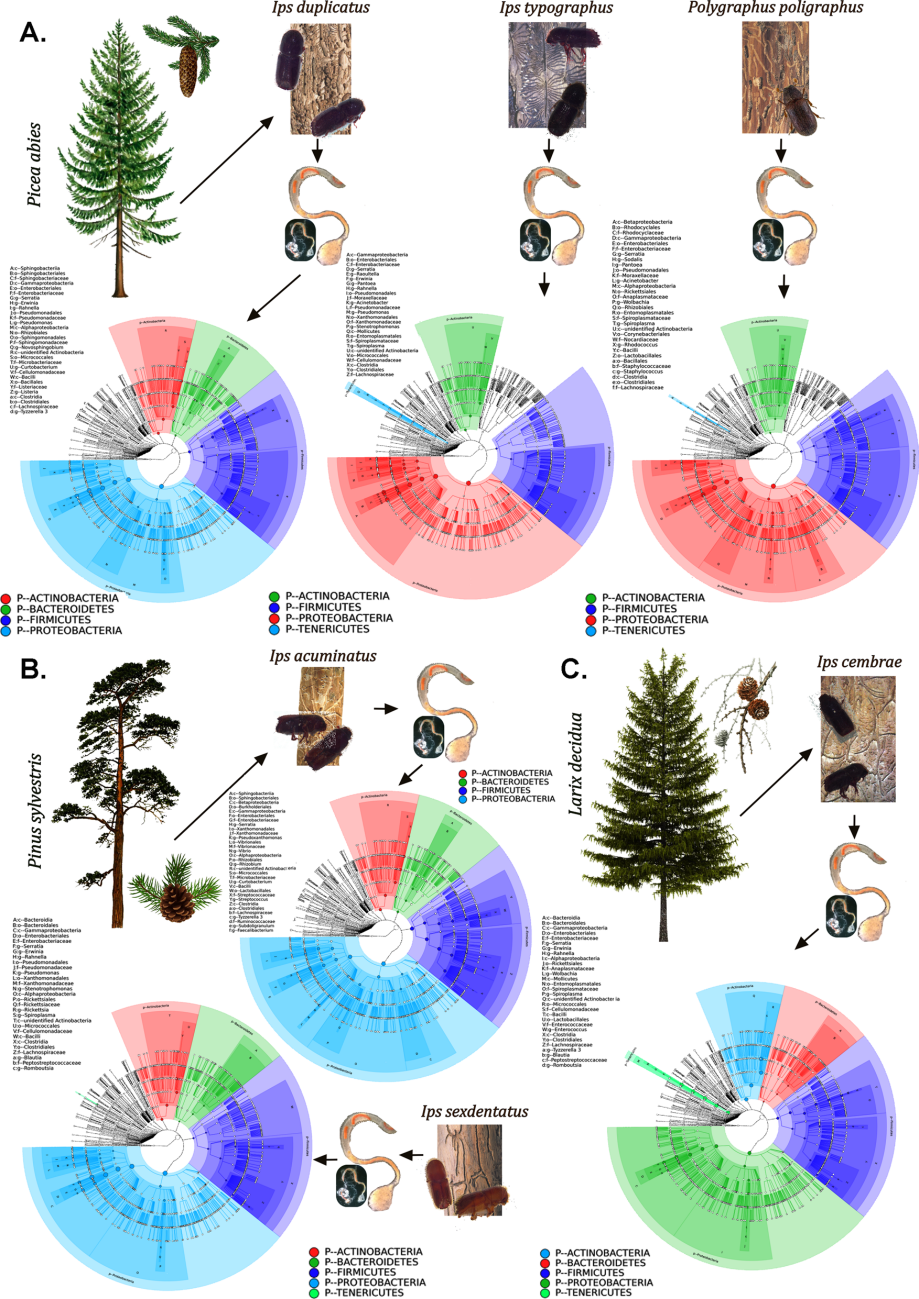
The gut bacterial abundance in different bark beetles represented using GraPhlAn. (**A**) *Ips duplicatus* (ID), *Ips typographus* (IT) and *Polygraphus poligraphus* (PP) feeding of spruce (*Picea abies*); (**B**) *Ips acuminatus* (IAC) and *Ips sexdentatus* (SX) feeding on pine (*Pinus sylvestris*) and (**C**) *Ips cembrae* (IC) feeding on larch (*Larix decidua*). The OTU trees represent the predominance of Proteobacteria, Firmicutes, Actinobacteria in five *Ips* and one non-*Ips* bark beetles. Bacteroidetes are observed in ID, IAC, SX, and IC. The circle illustrating the different taxonomic level range from inside out and the size of circles resemble the species abundance. Different colours stand for different phylum. The high abundance of top 40 species is denoted as solid circles.

**Figure 2:**
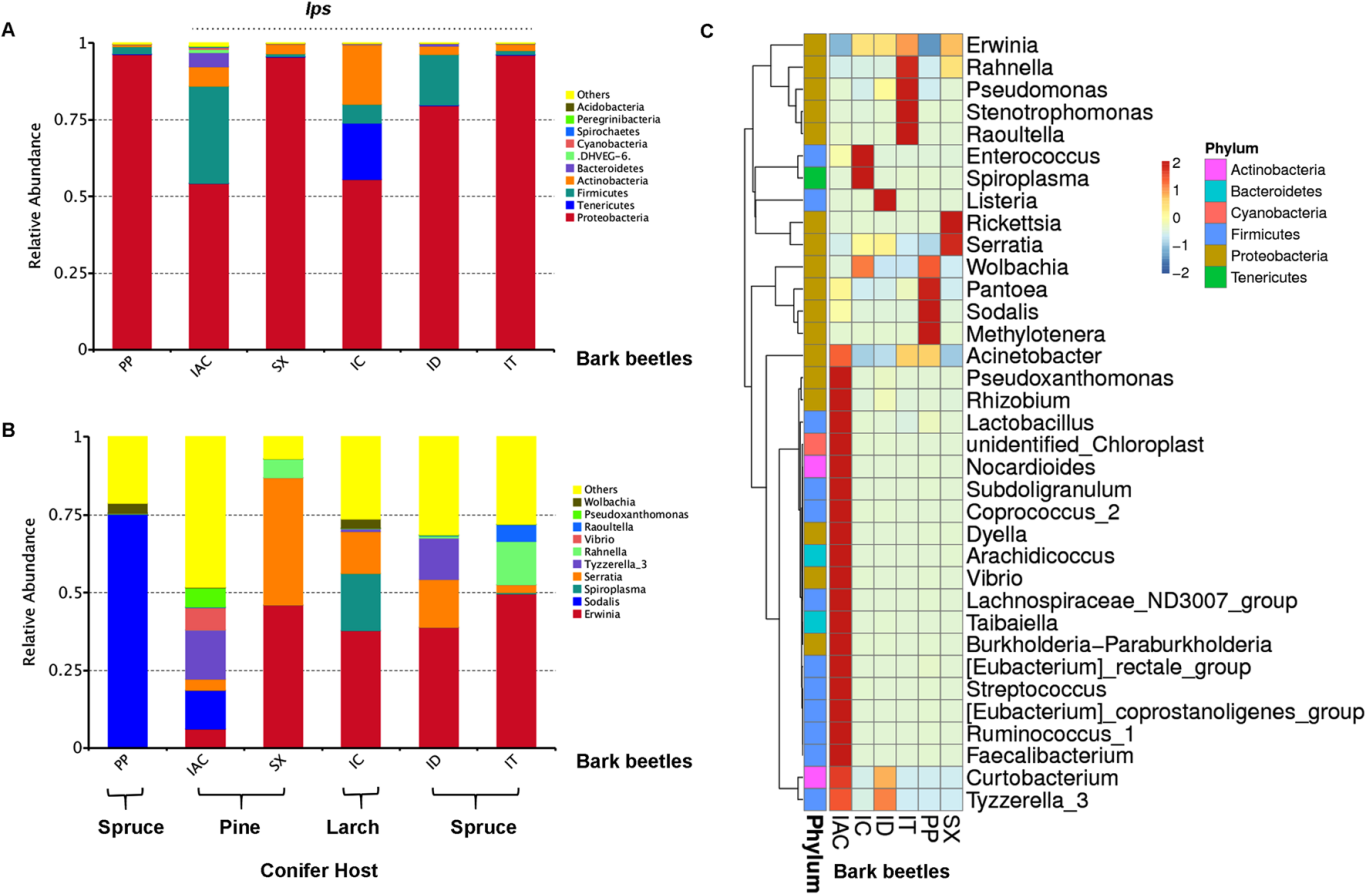
Bacterial diversity in bark beetle gut. (**A**) The bar plot represents the relative abundance of the gut bacteriome at the phylum level (top 10) depicting the dominance of Proteobacteria in five *Ips* and one non-*Ips* bark beetles. “Others” represents the total abundance of the rest of the phylum. (**B**) The relative abundance of top 10 bacterial genera present in the bark beetle gut. Similarly, “Others” represents the total abundance of the rest of the bacterial genera in the gut. (**C**) Heatmap showing the abundance of 35 dominant bacterial genera among five *Ips* and one non-*Ips* beetles. The colour gradient indicates the relative OTU abundance for each beetle where the darker colour denotes higher abundance, and the light colour represents low bacterial species abundance [*Ips typographus* (IT), *Ips duplicatus* (ID), *Ips sexdentatus* (SX), *Ips acuminatus* (IAC), *Ips cembrae* (IC) and *Polygraphus poligraphus* (PP)].

The bacterial sequences obtained from the gut tissue of five *Ips* and one non-*Ips* bark beetles were clustered into a total of 3049 OTUs at 97% similarity cut-off limit (Supplementary excel 3). The rarefaction curve and estimation of good’s-coverage indicating the completeness of sampling (> 99%) represent the whole bacterial diversity for each of the five *Ips* and one non-*Ips* bark beetle species that were covered (Supplementary Figure 1, Table 1). The sequences thus obtained were assigned to 43 bacterial phyla. Among these 43 bacterial phyla, the predominance of 5 bacterial phyla, namely Proteobacteria, Firmicutes, Actinobacteria, Bacteroidetes, and Tenericutes (Fig. 1) was represented using GraPhlAn. The relative abundance of the top 100 genera identified in this study was represented in the evolutionary tree as well as the top 10 genera was further denoted in the taxonomic tree (Supplementary Figure 2A,B). In particular, considering the top 10 phyla, the relative abundance of Proteobacteria was higher in all the beetle species (IT-96%, ID-79.6%, SX-95.4%, IAC-54.1%, IC-55.4% and PP-96.2%). However, IAC gut documented a relatively high abundance of Firmicutes (31.6%) and Bacteroidetes (4.7%) whereas Tenericutes (18.4%) and Actinobacteria (19.4%) were prevalent in IC (Fig. 2A and Supplementary excel 4). The most abundant (top 10) genera in bark beetle gut include *Erwinia*, *Sodalis, Serratia, Tyzzerella, Raoultella, Rahnella, Wolbachia, Spiroplasma, Vibrio,* and *Pseudoxanthomonas*. (Fig. 2B). Furthermore, the abundance heatmap representing 35 dominant bacterial genera includes the presence of additional members such as *Pseudomonas, Stenotrophomonas, Enterococcus, Listeria, Rickettsia, Pantoea, Acinetobacter, Methylotenera, Lactobacillus, Nocardioides, Lachnospiraceae* group*, Taibaiella, Burkholderia, Streptococcus, Ruminococcus, Faecalibacterium,* and *Curtobacterium* (Fig. 2C). To outstand the difference of dominant bacterial species within the bark beetles, ternary plots were constructed based on the relative abundance of the top 10 genera (Supplementary Figure 3). It was interesting to note that, within the spruce feeding *Ips* beetles, *Erwinia* was highly abundant in IT (49.6%), whereas *Serratia* predominated in ID (15.3%). While among the pine feeders, SX was dominated by both *Erwinia* (45.9%) and *Serratia* (40.9%), whereas IAC represented a high abundance of *Sodalis* (12.4%) and *Tyzzerella* (15.8%) in their gut. However, dominance of *Sodalis* (75.1%) is observed in non-*Ips* beetle (PP) while completely absent in other spruce-feeding *Ips* beetles (IT and ID). *Spiroplasma* (18.4%) and *Enterococcus* (4.1%) were observed to be high in the larch feeding beetle, IC compared to others (Supplementary Figure 3 and Supplementary excel 4).

**Table 1:** Alpha diversity indices.

Samples	Good’s coverage (%)^a^	Observed species^a^	ACE^a^	Chao1^a^	Shannon^a^	Simpson^a^
*Ips acuminatus* (IAC)	99.5	680.2 ± 38.4	831.3 ± 42.8	845.1 ± 75.7	4.8 ± 0.6	0.8 ± 0.1
*Ips sexdentatus* (SX)	99.8	139.0 ± 3.4	231.0 ± 10.2	222.1 ± 11.3	1.8 ± 0.2	0.6
*Ips cembrae* (IC)	99.7	217.3 ± 16.6	381.7 ± 27.3	341.2 ± 23.6	2.3 ± 0.2	0.7
*Ips duplicatus* (ID)	99.7	231.8 ± 10.1	373.4 ± 11.8	346.2 ± 11.8	2.3 ± 0.2	0.6
*Ips typographus* (IT)	99.8	171.5 ± 9.5	296.3 ± 23.9	274.8 ± 22.6	1.7 ± 0.4	0.4 ± 0.1
*Polygraphus poligraphus* (PP)	99.4	489.8 ± 42	736.2 ± 45.9	681.9 ± 43.8	1.9 ± 0.2	0.4 ± 0.1

^a^Data representing the mean value ± SE of six biological replicates for each bark beetle species. SE denotes standard error.

#### α-diversity

**Figure 3:**
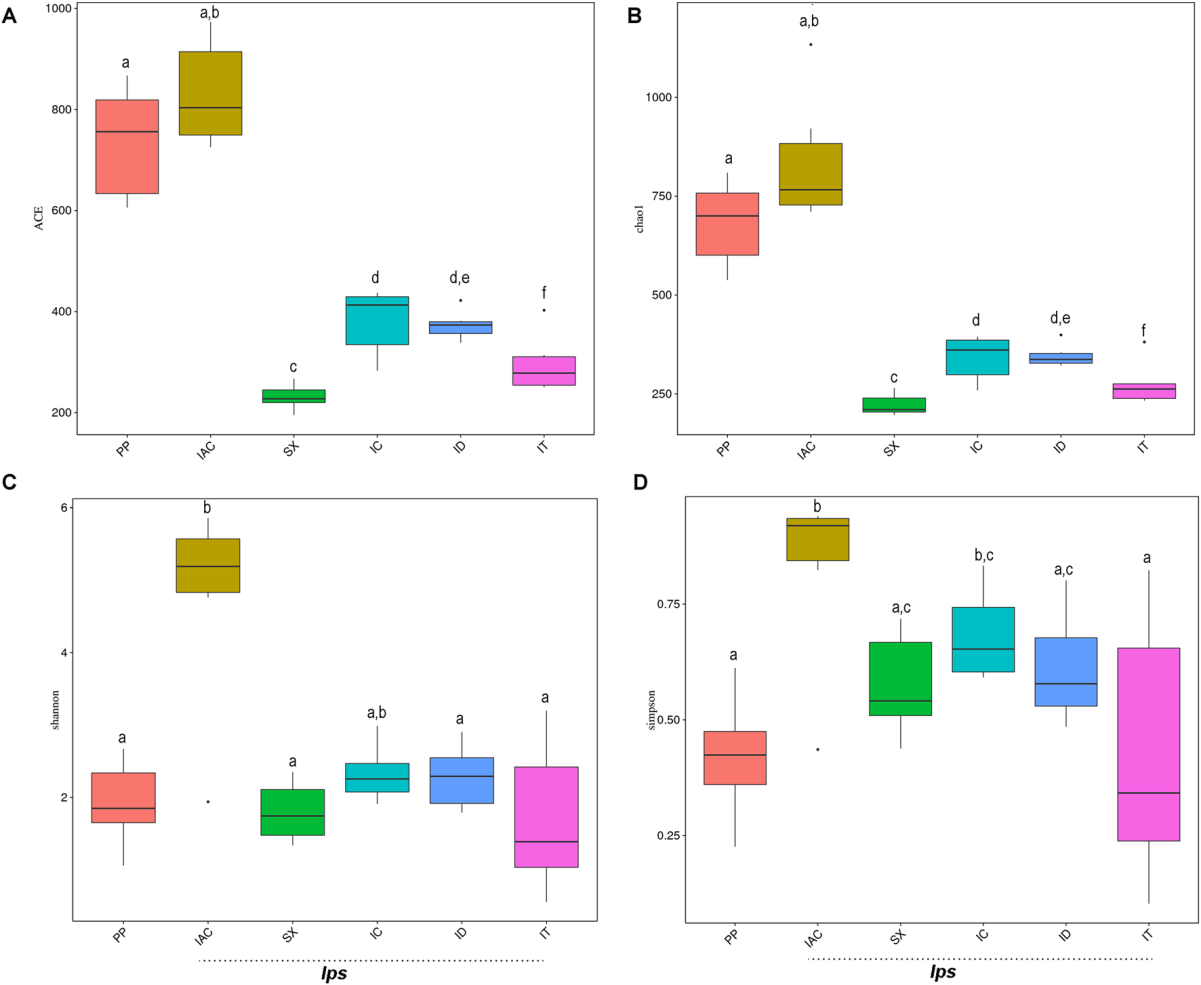
Boxplot illustrating the α-diversity indices between five *Ips* and one non-*Ips* bark beetles. The bacterial species richness indicated by (**A**) ACE analysis and (**B**) Chao1 analysis shows significant differences between the beetles feeding on different tree hosts. The bacterial diversity represented by (**C**) Shannon index and (**D**) Simpson index shows no significant variation among the spruce feeding *Ips* (ID, IT) and non-*Ips* beetles (PP) but the pine feeding *Ips* beetles (IAC, SX) differs significantly between them. Wilcoxon signed-rank test is performed for the analysis of the significance of the difference between groups. The same alphabets denote no significant differences. The figure is prepared using R software (Version 2.15.3; R Core Team, 2013, Vienna, Austria)^101^ [*Ips typographus* (IT), *Ips duplicatus* (ID), *Ips sexdentatus* (SX), *Ips acuminatus* (IAC), *Ips cembrae* (IC) and *Polygraphus poligraphus* (PP)].

The α-diversity indices illustrating the microbial community richness (ACE and Chao1; Wilcoxon signed-rank test) and diversity (Shannon and Simpson index; Wilcoxon signed-rank test) within each of the six bark beetle species (Fig. 3 and Table1). The bacterial richness among the spruce feeding *Ips* beetles was lower in IT (Chao1—274.8 ± 22.6; ACE—296.3 ± 23.9) and ID (Chao1—346.2 ± 11.8; ACE-373.4 ± 11.8) (P < 0.001) compared to non-*Ips* PP (Chao1—681.9 ± 43.8; ACE—736.2 ± 45.9). While, within the pine feeders, IAC (Chao1—845.1 ± 75.7; ACE—831.3 ± 42.8) showed significantly higher richness compared to SX (Chao1-222.1 ± 11.3; ACE-231.0 ± 10.2) (P < 0.001). Moreover, notable differences in gut-associated bacterial community diversity among the beetle species were also documented in the present study. IAC feeding on pine trees (Shannon index—4.8 ± 0.6; Simpson index—0.8 ± 0.1) showed significant differences (P < 0.05) compared to the spruce feeding *Ips* beetles (IT, ID) indicating the influence of different host feeding on shaping up the gut-associated bacteriome (Fig. 3 and Table1). However, the extent of bacterial diversity may vary from beetle species to species. Besides, the influence of host feeding, the significant difference (P < 0.01) between the pine feeding beetles IAC (Shannon index—4.8 ± 0.6; Simpson index—0.8 ± 0.1) and SX (Shannon index—1.8 ± 0.2; Simpson index—0.6) may also depict the environmental impact on the gut bacteriome. Furthermore, the number of observed bacterial species was higher in IAC (680.2 ± 38.4) compare to the other *Ips* bark beetles collected from R site (IT, ID, IC, and SX) (Table 1) indicating the plausible location-specific influence on gut bacterial richness but this needs further experimental validation.

**Figure 4:**
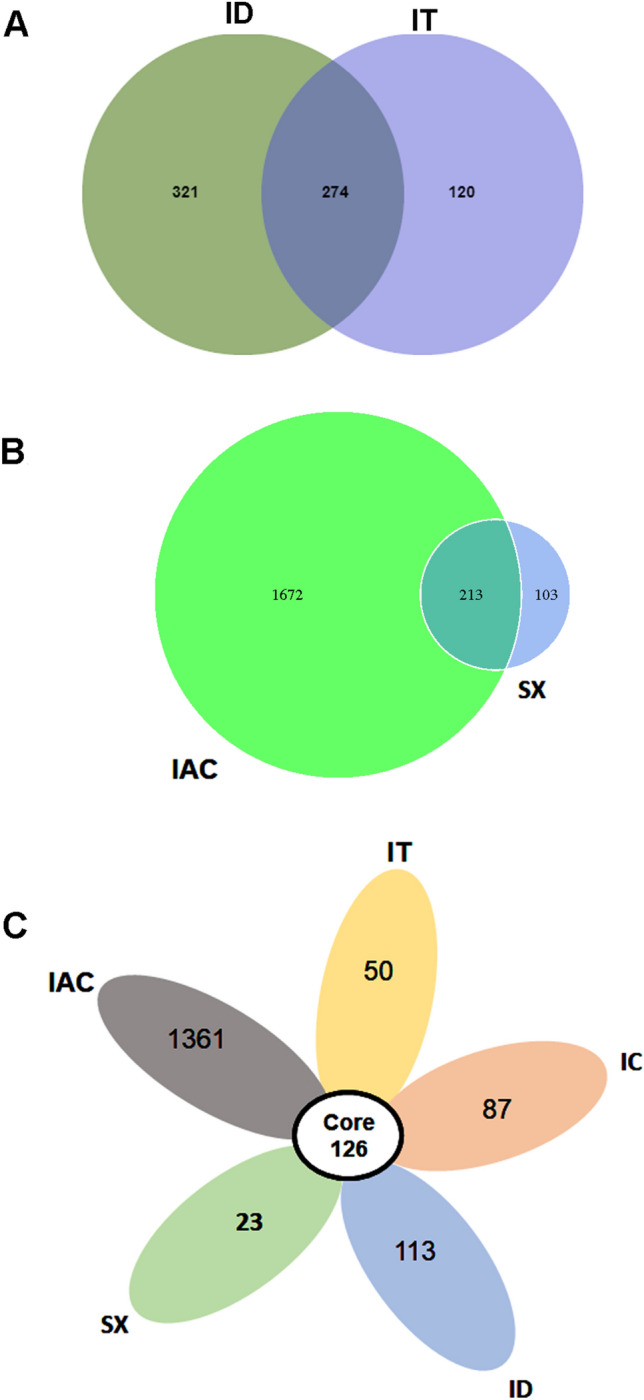
Core bacteriome. (**A**) Venn diagram showing the distribution of the bacterial OTUs among the spruce feeding *Ips* bark beetles (IT and ID) where the number of unique and common OTUs shared among the beetles is denoted. (**B**) Venn diagram representing the common and unique OTUs between the pine feeding *Ips* bark beetles (IAC and SX). (**C**) Core bacteriome among all five *Ips* species [*Ips typographus* (IT), *Ips duplicatus* (ID), *Ips sexdentatus* (SX), *Ips acuminatus* (IAC) and *Ips cembrae* (IC)].

Considering the *Ips* bark beetles feeding on spruce (IT, ID), 274 core OTUs were observed, whereas the pine feeding bark beetles from two different sites shared 213 OTUs in common (Fig. 4A,B and Supplementary excel 5, 6). Furthermore, the present study documented the occurrence of 126 core OTUs shared among all five *Ips* species (IT, ID, IC, SX and IAC) (Fig. 4C) that were assigned to 44 families and 69 genera. The shared community was primarily dominated by a consortium of 7 families (*Enterobacteriaceae, Lachnospiraceae, Moraxellaceae, Pseudomonadaceae, Ruminococcaceae, Sphingomonadaceae,* and *Xanthomonadaceae*) mainly constituting of *Erwinia, Serratia, Raoultella, Rahnella*, *Pantoea, Tyzzerella, Blautia, Roseburia, Lachnoclostridium, Acinetobacter, Psychrobacter, Pseudomonas, Ruminiclostridium, Oscillibacter, Faecalibacterium, Sphingobacterium, Sphingomonas, Pseudoxanthomonas*, *Stenotrophomonas* (Fig. 4C, Supplementary excel 7). Apart from the core bacterial community present in the bark beetle gut, a consortium of different unique OTUs was also detected. However, it is important to note that unique OTUs do not always refer to the unique bacterial species; nonetheless, the diversity of the unique bacterial species follows a similar trend in the present study (Supplementary excels 5,6,7).

#### β-diversity

**Figure 5:**
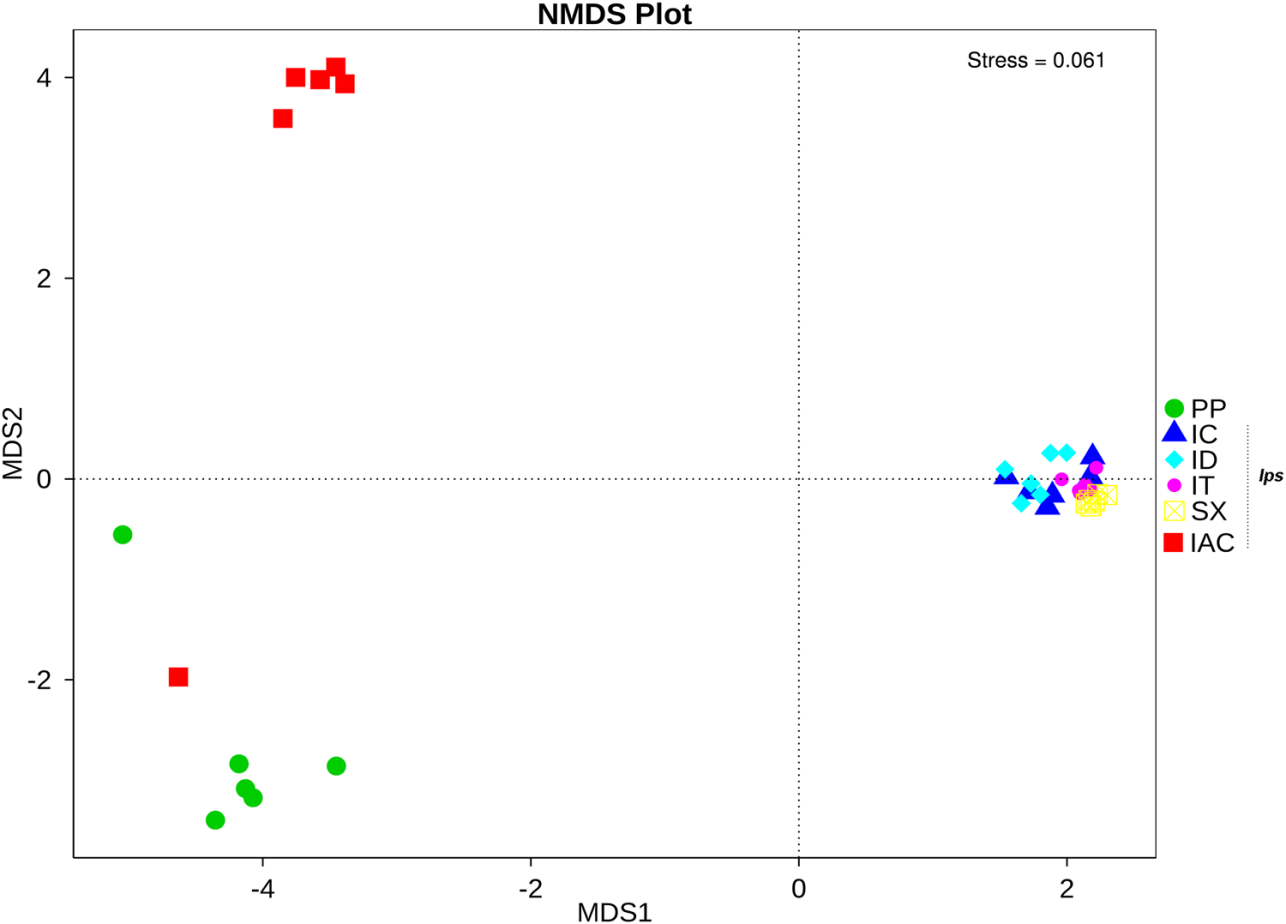
Non-metric multidimensional scaling analysis (NMDS)^102^ reflecting the extent of variation in the gut bacterial communities in the bark beetles. The data points in the same colour represent the same bark beetle species. Different symbols denote the different bark beetles. The *Ips* bark beetles collected from R-site (IT, ID, IC, SX) are clustered together showing no significant variation in their gut bacteriome but are different from *Ips* beetle collected from L-site (IAC) and non-*Ips* bark beetle collected from K-site (PP) [*Ips typographus* (IT), *Ips duplicatus* (ID), *Ips sexdentatus* (SX), *Ips acuminatus* (IAC), *Ips cembrae* (IC) and *Polygraphus poligraphus* (PP)].

Beta (β) diversity evaluating the differences in microbial communities between the bark beetles feeding on the different coniferous hosts was represented by the box plot and heatmap based on Unweighted UniFrac and Weighted UniFrac distances between samples (Supplementary Figures 4, 5A). The hierarchical clustering based on Unweighted UniFrac distance matrix (UPGMA—Unweighted pair group method with arithmetic mean) illustrates the influence of environment on gut bacterial diversity where all *Ips* beetle species collected from R site (IT, ID, IC, and SX) were clustered together in one clade (Supplementary Figure 5B). Similarly, Non-Metric Multi-Dimensional Scaling (NMDS) result also showed the clustering of the beetles together from the same site regardless of feeding on different conifer hosts suggesting the likely impact of sampling location on bark beetle gut microbial community (Fig. 5). Conversely, Weighted UniFrac UPGMA tree clustered all three spruce feeding beetles together irrespective of their location of sampling (Supplementary Figure 5C). Hence, no generalization can be derived at this point regarding the degree of influence sharing between the host plant and the location of bark beetle sampling in shaping up the gut bacteriome.

**Figure 6:**
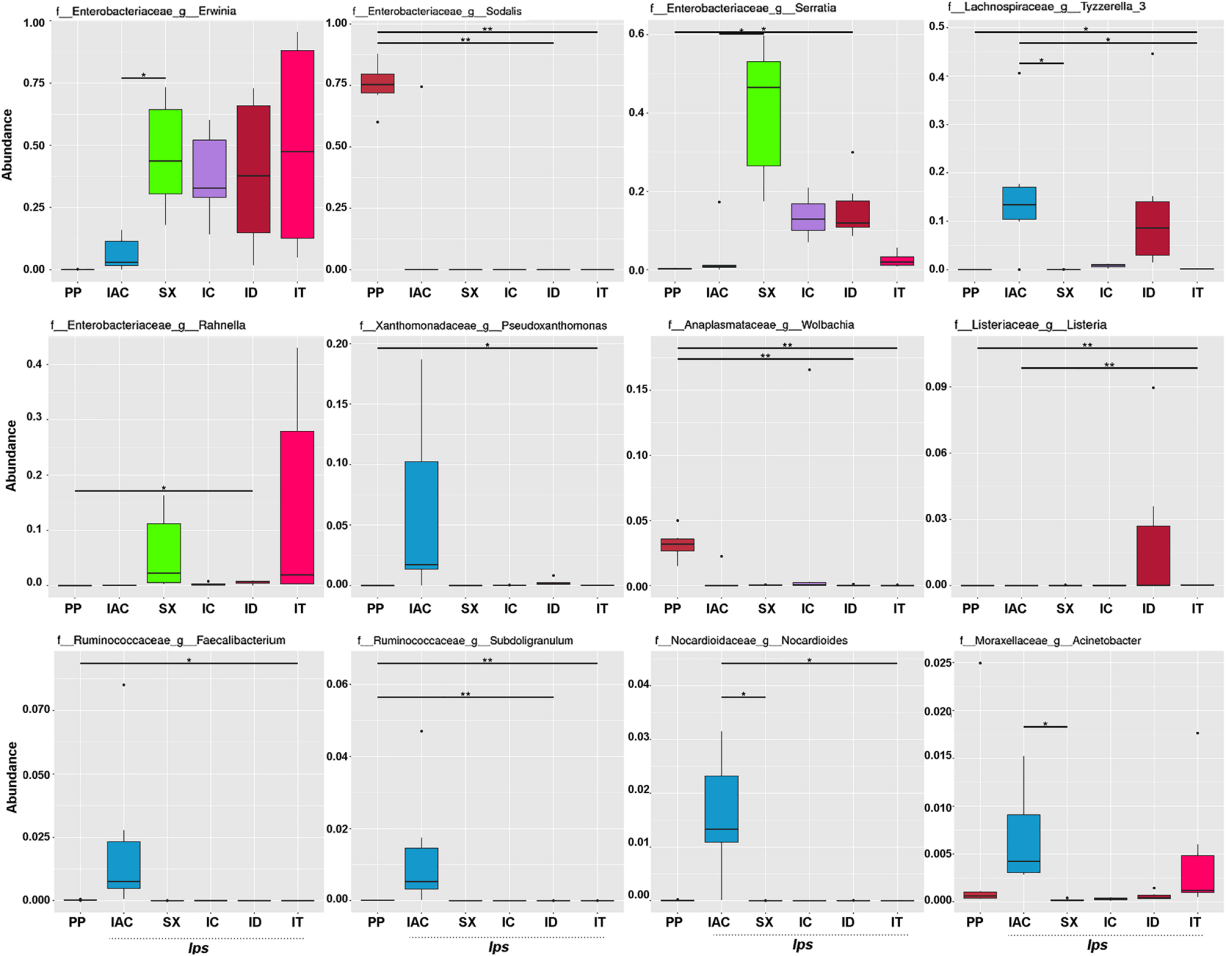
Metastats analysis showing the significant abundance of bacterial species within five *Ips* and one non-*Ips* bark beetles, where the FDR test evaluates the significance of observed abundance’s differences among beetles. The horizontal line represents the two groups with significant variation. “*” represents significant variation at q value < 0.05 while “**” denotes high significance at q value < 0.01 [*Ips typographus* (IT), *Ips duplicatus* (ID), *Ips sexdentatus* (SX), *Ips acuminatus* (IAC), *Ips cembrae* (IC) and *Polygraphus poligraphus* (PP)].

The extent of the top 12 bacterial species-specific variations at the genus level (Metastat analysis) revealed the predominance of *Erwinia* in *Ips* bark beetles collected from R-site (IT, ID, IC, and SX) while *Sodalis* is highly abundant in non-*Ips* PP collected from K-site. Considering the spruce feeding *Ips* beetles, the high abundance of *Rahnella* and *Acinetobacter* was detected in IT, whereas *Listeria* and *Tyzzerella* were abundant in ID. However, among the pine feeding bark beetles, *Tyzzerella, Pseudoxanthomonas, Faecalibacterium, Subdoligranulum, Nocardioides,* and *Acinetobacter* were most abundant in IAC whereas *Serratia* and *Rahnella* dominate in the gut of SX (Fig. 6). Moreover, t-test analysis performed to compare species-specific variation between the spruce feeding *Ips* bark beetles IT and ID from the same collection site revealed a significant difference in abundance of *Serratia* and *Pantoea* (Supplementary Figure 6). Furthermore, ANOSIM analysis evaluating the variation between the bacterial community among the six different bark beetles was significantly higher than the variation within the group (Supplementary Table 1). Our data revealed that the bacterial communities present in five *Ips* and one non-*Ips* bark beetle species are significantly different. However, MRPP and ADONIS analysis determining significant differences of the overall gut bacteriome among the bark beetles showed no such differences between IT and ID, both collected from R site and feeding on the spruce (Supplementary Tables 1, 2). In contrast, AMOVA analysis documented no significant differences between IT and SX (Supplementary Table 3) indicating marginally significant differences need to be interpreted carefully.

**Figure 7:**
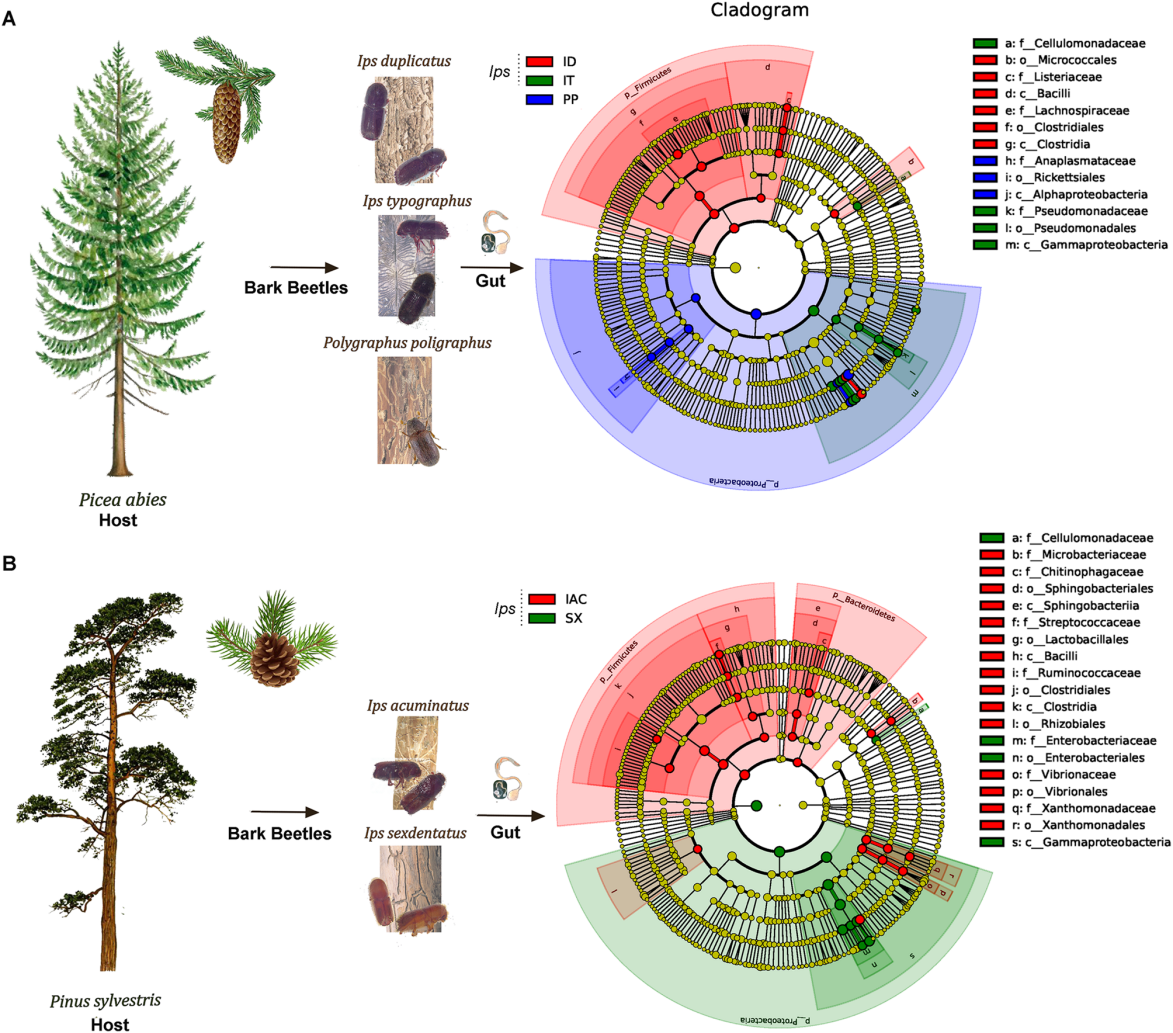
LEfSe analysis indicating the differentially represented bacterial biomarkers in the bark beetles. (**A**) The cladogram describes the presence of bacterial communities that significantly differs between the spruce feeding *Ips* and non-*Ips* bark beetles (IT, ID and PP). (**B**) The cladogram is illustrating the bacterial biomarkers in pine feeding *Ips* bark beetles (IAC and SX). The circles radiating from inside to outside designates the taxonomic level from phylum to genus. Each circle represents a distinct taxon at the corresponding taxonomic level. The size of each circle is proportional to the relative abundance of each taxon. Bacterial species (biomarkers) with significant differences are coloured according to the colour of corresponding bark beetle, whereas yellowish-green circles resemble non-significant bacterial species. Red and green nodes denote that these bacteria contribute highly to the group. Letters above the circles describe the bacterial biomarker [*Ips typographus* (IT), *Ips duplicatus* (ID), *Ips sexdentatus* (SX), *Ips acuminatus* (IAC), and *Ips cembrae* (IC)].

Linear discriminant analysis effect size (LEfSe) indicated the differentially abundant bacterial species between the bark beetles. The histogram of the LDA (Linear discriminant analysis) scores represented the predominance of the gut bacterial community of beetles feeding on different conifer hosts. Bark beetle species were characterized by a preponderance of some of the following significantly abundant genera (LDA score [log10] > 4) such as *Erwinia, Rahnella, Raoultella, Pseudomonas, Stenotrophomonas* in IT*; Serratia* in ID*;* Actinobacteria*, Spiroplasma, Enterococcus* in IC; *Serratia, Rahnella, Rickettsia* in SX*; Tyzzerella, Vibrio, Pseudoxanthomonas, Curtobacterium, Streptococcus, Faecalibacterium* in IAC while *Sodalis, Wolbachia, Pantoea, Enterobacter* in PP (non*-Ips*). (Supplementary Figure 7A). Considering all six bark beetles together, the presence of 1 to 7 bacterial biomarkers per beetle species was observed (Supplementary Figure 7B). Furthermore, within the spruce *Ips* feeding beetles (IT, ID) members from Proteobacteria (class—α-Proteobacteria and γ-Proteobacteria) and Firmicutes (class—Clostridia and Bacilli) served as predominant biomarkers. Similarly, bacterial species from the phylum Bacteroidetes (class—Sphingobacteriia) was also prevalent in pine feeding beetle along with Firmicutes and Proteobacteria. In particular, *Lachnospiraceae*, *Cellulomonadaceae*, Pseudomonadales, Clostridiales, Bacilli, Micrococcales were observed as important biomarkers in spruce feeding *Ips* beetles (Fig. 7A, Supplementary Figure 8A). While *Microbacteriaceae*, *Chitinophagaceae*, Rhizobiales, *Streptococcaceae*, *Ruminococcaceae*, Sphingobacteriales, Xanthomonadales, Vibrionales, *Cellulomonadaceae*, and Enterobacteriales were prevalent markers for the pine feeding bark beetles (IAC and SX) (Fig. 7B, Supplementary Figure 8B).

#### Functional diversity

**Figure 8:**
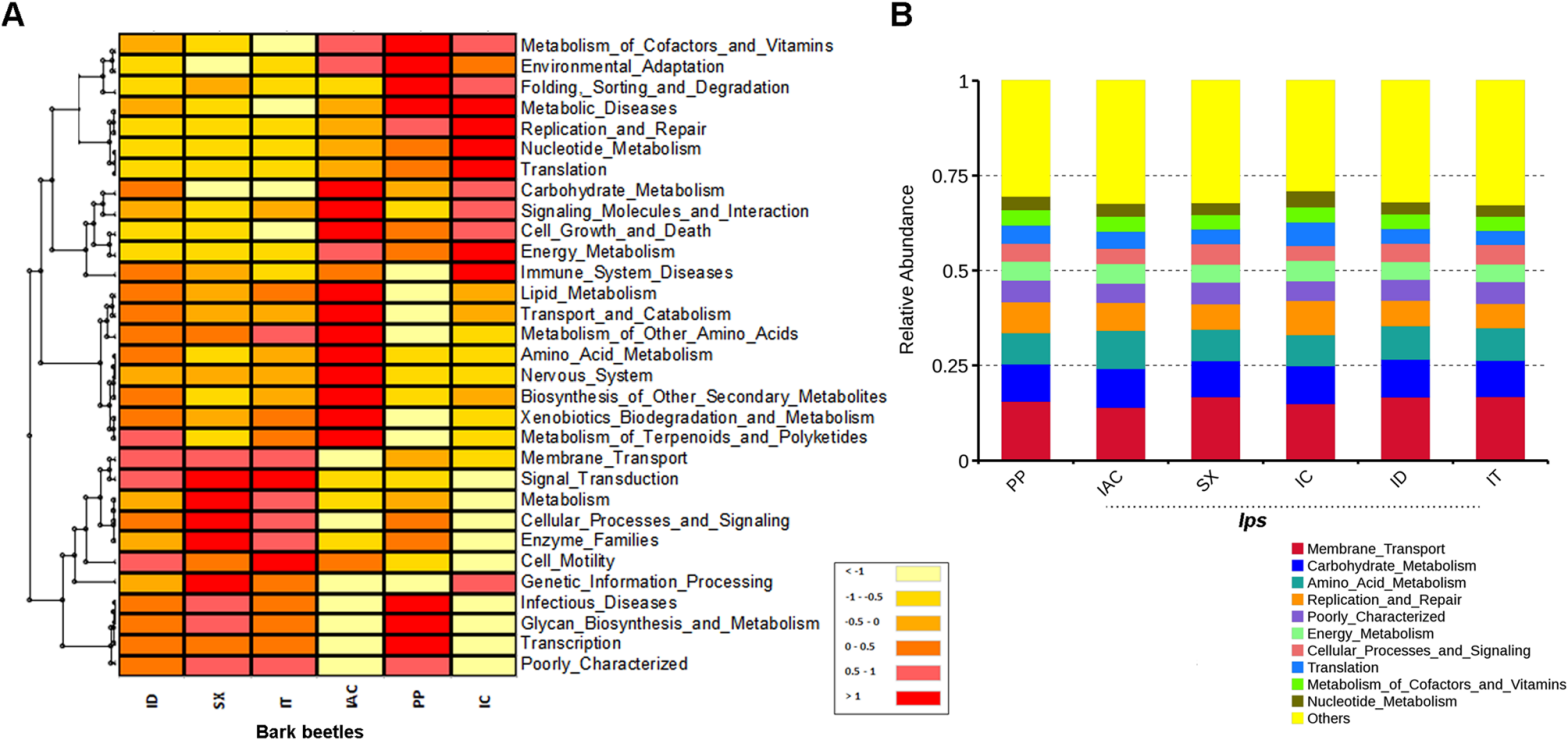
Functional prediction using PICRUSt. (**A**) Heatmap illustrating the functional profile predicted at level 2 KEGGs Orthologs using PICRUSt analysis represents the overall functional contribution of gut bacterial communities present in five *Ips* and one non-*Ips* bark beetles. (**B**) Barplot describing the relative OTU abundance contributing to the top 10 gene functions in the bark beetles shows no significant differences referring to the role of the core bacteriome. “Others” represent the relative OTU abundance for the rest of the gene functions [*Ips typographus* (IT), *Ips duplicatus* (ID), *Ips sexdentatus* (SX), *Ips acuminatus* (IAC), *Ips cembrae* (IC) and *Polygraphus poligraphus* (PP)].

The putative functional profile of the bacterial community based on the relative abundance of the marker gene (16S) sequences was documented using PICRUSt. The heatmap cluster illustrates the functional diversity of the bacterial communities’ present in the beetles where the contribution of each OTU is associated with a given gene function. The relative abundance of the following gene functions such as carbohydrate metabolism, cell growth and death, immune-related function, lipid metabolism, metabolism of Terpenoids and Polyketides, biodegradation and metabolism of xenobiotics, biosynthesis of other secondary metabolites, amino acid metabolism, transport and catabolism, signalling of molecules and their interaction, revealed at level-2 KEGGs Orthologs (KOs), were highly represented in IAC. The genes involved in metabolic diseases, replication, and repair, translation, nucleotide metabolism, immune response, energy metabolism was dominant in larch feeding beetle IC (Fig. 8A). However, considering the relative abundance of the top 10 gene functions, no significant difference was observed among different *Ips* bark beetles (Fig. 8B). Such profile of highly abundant gene function such as membrane transport, carbohydrate metabolism, amino acid metabolism, replication and repair, cellular processes and signalling, translation, metabolism of cofactors and vitamins and nucleotide metabolism might be attributed to core bacterial communities in *Ips*, which may contribute to the fundamental functions in bark beetle physiology. Comparing with *Ips* and non-*Ips* bark beetles, the genes involved in metabolic diseases, degradation, environmental adaptation, metabolism of cofactors and vitamins, transcription, glycan biosynthesis, and metabolism and immune function were relatively less abundant within *Ips* bark beetles.

## Discussion

Bark beetles serve as a keystone species in forest ecosystems by nutrient cycling of mature and wind-felled trees. However, these beetles become a severe pest for production forests during the outbreak phase, causing considerable economic as well as environmental damage. The present study characterizes the gut-associated bacterial communities of five *Ips* and a non-*Ips* bark beetles from the Scolytinae subfamily feeding on different conifer hosts. The study reveals the core bacterial communities in the gut of two spruce feeding, two pine feeding, and one larch feeding *Ips* bark beetles collected from two forest locations within Czech Republic and their putative functional relevance in bark beetle holobiont.

In our study, the bacterial community structure reflected by the α-diversity indices illustrates lower bacterial diversity and richness in *Ips typographus* (IT), *Ips sexdentatus* (SX), while *Ips acuminatus* (IAC) exhibited highest richness and diversity among all *Ips* bark beetles. ID and IC displayed similar bacterial diversity and richness that is somewhat in between IT, SX and IAC (Fig. 3). The higher diversity of bacteriome in IAC may also be co-related with their fungi feeding behaviour during larval stages^53^. High bacterial diversity may be obligatory to support such feeding habit. Furthermore, IAC being a less aggressive beetle compare to *Ips sexdentatus* considering their ophiostomatoid fungi arsenal^50^, may also need a wide array of bacterial species to cope with the pine defensive compounds. However, considering the attack behaviour in the forest IAC can attack healthier trees than SX^54^, which is considered as typical secondary pest^55^. Alternatively, *Ips typographus* is considered as aggressive beetle species that execute a mass attack to overwhelm the plant defence mechanism and thereby can sustain well with low bacterial diversity in their gut. However, such inferences would need further experimental validation where both aggressive and less aggressive bark beetle species need to be collected from a particular location, feeding on the same host tree to nullify the influence of microenvironments and the differences in feeding habitat. Besides, identification of the host tree microbiome would also be informative in this context.

Bark beetle gut bacterial assemblage is a non-random process^56^. However, several factors such as beetle sampling location, population characteristics (epidemic or endemic), competition for survival and resource acquisition, host quality or availability, and trophic interactions within the microhabitat can directly or indirectly influence the bacterial community at a given time in the bark beetle gut. Despite all these sources of variabilities, our results document the presence of a core bacteriome representing nine phyla identified to 44 bacterial families and 69 bacterial genera across all five *Ips* bark beetles (Fig. 4C). It can be presumed that the obtained core microbiome involved in conserved function in bark beetles. The data represents the predominance of Proteobacteria followed by Firmicutes, Actinobacteria, and Bacteroidetes that are consistent with the previous studies done on other bark beetles from the same subfamily^57^. To colonize and survive under the tree bark, the bark beetles largely depend on their gut bacterial species to digest the complex celluloses, hemicelluloses, and lignin to provide nutrition to the beetles^31,33^. In the current study, bacterial genera, namely *Erwinia, Serratia, Rahnella, Raoultella,* and *Pantoea* belonging to *Enterobacteriaceae* family, frequently observed in the core bacteriome in *Ips* bark beetles may contribute to such conserved function to survive in the similar habitat under the bark^57^. Besides, *Pseudomonas, Stenotrophomonas, Pseudoxanthomonas, Novosphingobium, Sphingomonas* and species from *Ruminococcaceae* family also present among the core microbiome are reported to degrade cellulose and other plant cell wall polysaccharides such as starch, xylan, and lignin, and the breakdown products derived may provide nutrition to beetles^31,33,34,58,59^. Furthermore, *Acinetobacter* detected in our study demonstrate esterase and lipolytic activity^60^. Such abilities of the bacterial species to hydrolyze the phospholipids, triglycerides, and fatty acids present in the phloem and resin of the tree^61^ may contribute to energy reserve in the fat bodies of bark beetles that can be utilized later during beetle development as well as host colonization and reproduction^62,63^.

Handling the nitrogen limitation in the diet is a communal challenge for all bark beetles feeding on a nitrogen-limiting phloem diet. Bark beetles often rely on their gut symbiotic bacteria capable of nitrogen acquisition for mitigating the crisis^64^. Nitrogen-fixing bacteria belonging to genera *Rahnella, Stenotrophomonas, Pantoea, Raoultella, Pseudomonas*, *Burkholderia,* and *Bradyrhizobium,* which are present as the core bacteriome may be abetting *Ips* bark beetles out of the crisis^65–68^. Moreover, *Pseudomonas* and *Rahnella* can recycle the uric acid from the beetle faeces into assimilable nitrogen, aiding bark beetle sustenance^65,66^. For successful tree colonization, the bark beetles also face challenges to combat the host defensive compounds that are released in high amounts soon after bark beetle attack^69^. Though such compounds at low levels enable the bark beetles in finding their suitable host^5^, the same host defence compounds in high concentration (i.e., terpenoids) are entomotoxic. To outcompete the plant defence mechanisms, some bark beetles follow different strategies such as overwhelming the tree defence by a mass attack or by surviving in the hostile environment with assistance from their symbionts^16^. *Pseudomonas, Serratia, Rahnella, Erwinia,* and *Pantoea* identified as core members of *Ips* bark beetle gut in the present study may help in the deprivation of toxic monoterpenes in addition to degradation of the plant cell wall polysaccharides and nitrogen fixation in those beetles^35,36,41^. Moreover, bacterial symbionts are often implicated in providing vitamins and amino acids to insect host^20,70,71^. Some bark beetles ingest fungi that supplement the beetles with vitamins, amino acids, and sterols^27,29^. Interestingly, bacterial species *Pseudomonas* is reported to produce a variety of antifungal antibiotics to prevent the growth of pathogenic fungi in *Dendroctonus* genus^72,73^, whereas *Stenotrophomonas* and *Pantoea* are documented to function against the pathogen in red turpentine beetles^66^. Additionally, *Clostridiaceae* observed in the core bacterial community is also reported to produce antimicrobial molecules, but their function is yet to be discovered^74^. Furthermore, *Pseudomonas, Serratia, Rahnella, and Erwinia* that are detected in the present study is reported to be associated with the production of bark beetle anti-aggregation pheromone, verbenone^40^. Plant defensive compound such as α-pinene serves as a precursor of verbenone, a common bark beetle anti-aggregation pheromone^75,76^. The biochemical pathway of verbenone production from cis-verbenol is recently documented to be mediated by enzymes originated from facultative anaerobes in *D. valens* gut (red turpentine beetle) under the frass-simulated and gut-simulated oxygen concentration environment^77^. Our present findings also endorse such possibilities; however, that requires further investigation.

Our data propose that the *Ips* bark beetles share a persistent consortium of core bacterial communities that may contribute to the fundamental metabolic pathways such as cellulose degradation, nitrogen fixation, detoxification of defensive plant compounds, and inhibition of pathogens. However, the variation in the gut bacterial communities among the bark beetles, observed from the β-diversity, maybe due to demographic factors that comprise the discrepancies in feeding habitat and other environmental characteristics besides the prevailing difference at the species level. The environmental influence (i.e., location of sampling) on the bark beetle ecology plays a vital role in shaping the gut bacteriome. Interestingly, the *Ips* bark beetles collected from the R site (IC, SX, IT, and ID) are clustered together (Fig. 5) and show similar variations overall in their gut bacterial communities when compared to other *Ips* bark beetle from L-site (IAC) and non-*Ips* bark beetle (PP) collected from K-site. Such findings could be explained by the fact that several bacterial species are also acquired from feeding on the different coniferous hosts in the same forest environment. However, such conclusions need further experimental corroborations.

However, it is interesting to note that besides the core bacterial communities, the *Ips* bark beetles also host different bacterial genera in the gut, which could be assimilated during feeding on to the phloem tissue. The occurrence of *Spiroplama* belonging to Tenericutes is significantly higher in larch feeding beetle, IC (18.4%) compared to other beetles (< 0.5%) but completely absent in IAC. Previous studies reported *Spiroplasma* as an insect symbiont, but its role in the gut is yet to be identified^78^. Furthermore, *Pseudomonas*, *Serratia,* and *Pseudoxanthomonas*, present as core gut members in IC, are also detected in the cuticle and galleries of other larch feeding beetle, *Dendroctonus simplex* (eastern larch beetle)^79^. The presence of Actinobacteria in the gut of the IC may produce antimicrobial compounds. It is documented that the presence of *Streptomyces* belonging to Actinobacteria phylum is producing antifungal agents against the growth of *Ophiostoma minus* in *Dendroctonus frontalis* Zimmermann (southern pine beetle)^80^. Based on our knowledge, it is the first record for *Sodalis* to be present in the gut of bark beetles. However, *Sodalis* is only present in IAC (12.4%) and absent in other *Ips* beetles. Though the role of *Sodalis* in bark beetles is not yet understood completely, hitherto, it is described as a mutualistic endosymbiont in tsetse flies and heteropteran insects with extensive metabolic capabilities^81,82^. In addition to the above-mentioned bacterial species, the predominance of *Wolbachia* and *Rickettsia* are considered as a symbiotic pathogen associated with several beetle species for inducing cytoplasmic incompatibility, resulting in reproductive distortions and hence, may serve as candidates for future management of bark beetles^83,84^.

Noteworthy, each beetle species hosts a set of specific bacterial genus as biomarkers, and some of the bacterial biomarkers congregate in the functional profile of the bark beetles evident from the PICRUSt analysis (Fig. 8). Some of the biomarkers detected in the present study have the potential to play a vital fundamental role in bark beetle physiology hence can be the targets for further investigations. For instance, the presence of bacterial species belonging to the families *Lachnospiraceae, Cellulomonadaceae, Ruminococcaceae, Chitinophagaceae* is associated with degradation of complex plant polysaccharides by their cellulolytic enzymes^58,85^. In addition to the carbohydrate metabolism ability, some members of Enterobacterials and Pseudomonadales are also capable of nitrogen fixation, terpenoid degradation and metabolism of xenobiotic compounds^35,36,41,66^. The production of antimicrobial compounds to inhibit the growth of pathogens and contributing to the immune response of the bark beetles are attributed to the presence of bacterial biomarkers such as Pseudomonadales^72,73^. Moreover, the top 10 physiological functions contributed by gut bacterial community seems to be conserved among all *Ips* beetles, and that is perhaps associated with the core bacterial communities (Fig. 8B). However, such functional aids from the gut bacteria to the host beetles need to be experimentally validated.

In conclusion, it is a pioneering study surveying the gut-associated (endomicrobiome) bacterial community in five *Ips* bark beetle species inhabited in Czech forests. Sixty-nine core bacterial genera are documented and explored for their putative ecological functions. Our data advocates plausible conserved functions for the core gut-bacteriome in different *Ips* beetles irrespective of ecological and demographic variabilities among them. Hence, they can serve as targets for future downstream functional studies (i.e., metatranscriptomics and metaproteomics). It is essential to mention here that the in-depth knowledge about the core gut bacterial communities and their fundamental function in *Ips* bark beetle adaptation to survive in a hostile environment could expand our knowledge about the important forest nutrient recycler and may unleash the unexpected potential for keeping bark beetle population in the non-epidemic stage.

## Materials methods

### Sample collection, dissection, and DNA extraction

Emerged adult bark beetle samples were collected from infested trees from three forests in the Czech Republic during May and June 2018. Precisely, *Ips typographus* (IT), *Ips duplicatus* (ID), *Ips cembrae* (IC); *Ips sexdentatus* (SX) were collected from Rouchovany (49°04′08.0″ N 16°06′15.4″ E) (R-site; under State Forest Enterprise, public forest, regular forest management, warm and drought area ); *Ips acuminatus* (IAC) was obtained from Libavá (49°40′18.8″ N 17°31′44.1″ E) (L-site; Military Forest Enterprise, forest with restricted moving of people, regular forest management, Semi humid and slightly colder area compare to R site), and *Polygraphus poligraphus* (PP) was collected from Kostelec nad Černými lesy (50°00′07.2″ N 14°50′56.3″ E) (K-site; under School Forest Enterprise, close to nature forest management, forest near urban area, semi warm and dry area) in Czech Republic. Taxonomic identification of the beetles was made based on published literature by Pfeffer^86,87^ and Nunberg^88^. To assimilate six biological replicates per beetle species representing the beetle population in the area, more than 120 living and healthy beetles were collected and pooled from infested logs in each locality belonging to 8 or more different trees and subsequently shock frozen under liquid nitrogen for future use. It is noteworthy to mention here that due to the pooling of beetles during collection, the individual colony specific variability of the beetle gut microbiome, which was not the primary study goal, could not be estimated.

Randomly selected bark beetles were surface sterilized and dissected using dissecting scopes in the biosafety cabinet under sterile conditions^89^. Only guts that were free of apparent nematode infections were used for downstream DNA extraction. Subsequently, gut tissues (8 to 10 guts pooled per replicate) were homogenized, and microbial DNA was extracted using PureLink Microbiome DNA Purification Kit (Invitrogen, Thermo Fisher Scientific, US) following manufactures protocol. Purified DNA was quantified using Qubit 2.0 Fluorometer (Thermo Scientific) using Qubit dsDNA HS Assay kit (Invitrogen, Thermo Fisher Scientific, US), and the integrity was evaluated by 1% agarose gel electrophoresis. Lastly, extracted high-quality DNA from all beetle species (six biological replicates per species) was sent for high-throughput amplicon sequencing (Novogene Company, China).

### Amplicon sequencing

Amplicon sequencing was performed following the pre-optimized protocol. Precisely, DNA was diluted to 1 ng/μL using DNase free water, and bacterial 16S rRNA genes of distinct regions (V3-V4) were amplified using a specific primer (341F-806R)^90^ with the barcode. All PCR reactions were performed with Phusion High-Fidelity PCR Master Mix (New England Biolabs). PCR products were visualized on 2% agarose gel, and samples with clear amplification between 450 and 480 bp were selected for further experiments. PCR products were purified with the Qiagen Gel Extraction Kit (Qiagen, Germany). Sequencing libraries were generated using NEBNext Ultra DNA Library Pre-Kit for Illumina, and index codes were added. The library quantity and quality were measured on the Qubit 2.0 Fluorometer (Thermo Scientific) and Agilent Bioanalyzer 2100 system. Lastly, the library was sequenced on a Novaseq 6000 platform, and 250 bp paired-end reads were generated.

### Data analysis (α-diversity/ β-diversity and core microbiome/functional analysis/statistics)

#### Paired-end reads assembly and quality control

Paired-end reads were assigned to individual samples based on unique sample-specific barcodes and truncated by cutting off the barcode and primer sequences. Assembly of paired-end reads was done using FLASH (V1.2.7, https://ccb.jhu.edu/software/FLASH/)^91^. Quality filtering on the assembled sequences (raw tags) was performed to obtain high-quality clean tags setting pre-set parameters^92^ in QIIME (V1.7.0, https://qiime.org /index.html)^93^. Chimaeras were removed after comparing them with the reference database (i.e., Gold database) using the UCHIME algorithm^94^ to detect chimaera sequences, and subsequently, the chimaera sequences were removed, and the useful Tags were finally obtained.

#### OTU cluster and species annotation

Sequence analysis was achieved using UPARSE software (UPARSE v7.0.1001, https://drive5.com/ uparse/)^95^. Sequences with ≥ 97% similarity were allotted to the same OTUs, and the representative sequence for each OTU was screened for species annotation using SSUrRNA database of SILVA Database (https://www.arb-silva.de/)^96^ for species annotation at each taxonomic rank (Threshold: 0.8–1)^97^. To evaluate the phylogenetic relationship of different OTUs, multiple sequence alignment was performed using the MUSCLE software (Version 3.8.31,https://www.drive5.com/muscle/)^98^. Finally, OTU abundance was normalized using the sequence number corresponding to the sample with the least sequences. Downstream analysis of α and β diversity was performed using normalized abundance data.

#### α-Diversity

The complexity of species diversity (α-diversity) was estimated for each sample using standard indices such as observed-species, sequence depth (Good-coverage)^99^, community richness (Chao1, ACE)^100^, diversity (Shannon, Simpson)^100^. All these indices in beetle samples were calculated with QIIME (Version 1.7.0)^93^ and displayed using R software (Version 2.15.3; R Core Team, 2013, Vienna, Austria)^101^.

#### β-Diversity

Beta (β) diversity analysis was used to evaluate variances in species complexity in different beetle samples. Beta (β) diversity was calculated in QIIME software (Version 1.7.0)^93^ using weighted and unweighted unifrac distances to measure the dissimilarity coefficient between pairwise samples. Non-metric multidimensional scaling analysis (NMDS)^102^, a non-linear model designed for a better representation of non-linear biological data structure, is performed to get principal coordinates and visualize the complex, multidimensional data. Unweighted Pair-group Method with Arithmetic Means (UPGMA)^103^ Clustering was performed as a type of hierarchical clustering method to interpret the distance matrix using average linkage and was conducted by QIIME software (Version 1.7.0)^93^. Variation analysis of bacterial community structure between different beetle sample groups was evaluated by standard statistical methodologies such as Analysis of Similarity^104^ (ANOSIM, a nonparametric test to evaluate whether variation among groups is significantly larger than the variation within groups), Multi-response permutation procedure (MRPP)^105^ analysis (similar with ANOSIM, which aims at determining whether the difference of microbial community structure among groups is significant), ADONIS^106^ ( also called permutational MANOVA or nonparametric MANOVA, which is a method of nonparametric multivariate variance test according to the distance matrix, e.g., Bray–Curtis, Euclidean, etc.), AMOVA^107^ (similar to ADONIS, which is a kind of nonparametric method aiming at determining whether the difference of microbial community structure among groups is significant). Furthermore, variation analysis of bacterial species between the different beetle sample groups was estimated by T-test^108^ and Metastats^109^. Precisely, species with significant intra-group variation were detected via Metastats^109^, rigorous statistical methods based on bacterial abundance measurement. The significance of observed abundances differences among groups is further evaluated via multiple hypothesis-test for sparsely-sampled features and false discovery rate (FDR).

Lastly, LEfSe [linear discriminant analysis (LDA) Effect Size] analysis was performed to detect key bacterial species with a significant intra-group variation^110^ among tested beetle sample groups. Alternatively, LEfSe is a software aiming at discovering high-dimensional biomarkers and revealing metagenomic features, including genes, metabolic, or taxa, thus can be used to distinguish two or more biological classes. It emphasizes statistical significance, biological consistency, and effect relevance and allows us to identify features of abundance and related classes.

### Functional prediction using PICRUSt

Functional prediction of the metagenome was made using PICRUSt^111^ (Phylogenetic Investigation of Communities by Reconstruction of Unobserved States, version 1.0.0) using OTU table generated in QIIME software (Version 1.7.0)^93^. Kyoto Encyclopaedia of Genes and Genomes (KEGG)^112^ were used to calculate the predicted abundance of different gene families. PICRUSt output table was used to build a heat map.

## Supplementary information

Supplementary Information 1.

Supplementary Information 2.

Supplementary Information 3.

Supplementary Information 4.

Supplementary Information 5.

Supplementary Information 6.

Supplementary Information 7.

Supplementary Information 8.

## Data Availability

The datasets generated and/or analyzed during the current study are available under NCBI Bio-project PRJNA627871 (Bio-sample accession SAMN14689168–SAMN14689203).
