# Supplementary material for: Unravelling the gut bacteriome of Ips (Coleoptera: Curculionidae: Scolytinae): identifying core bacterial assemblage and their ecological relevance
Source: Sci Rep. 2020 Oct 29;10:18572. doi: 10.1038/s41598-020-75203-5 (PMC7596566; doi:10.1038/s41598-020-75203-5)

Unravelling the gut bacteriome of *Ips* (Coleoptera: Curculionidae: Scolytinae): identifying core bacterial assemblage and their ecological relevance.

Amrita Chakraborty[1], Muhammad Zubair Ashraf[2], Roman Modlinger[2], Jiří Synek[2], Fredrik Schlyter[2,3], Amit Roy [2#]

Supplementary Materials

Affiliations:

[1] EVA 4.0 Unit, Faculty of Forestry and Wood Sciences, Czech University of Life Sciences Prague, Kamýcká 129, CZ – 165 21 Praha 6 – Suchdol, Czech Republic.
[2] Excellent Team for Mitigation (ETM), Faculty of Forestry and Wood Sciences, Czech University of Life Sciences Prague, Kamýcká 129, CZ – 165 21 Praha 6 – Suchdol, Czech Republic.
[3] Department of Plant Protection Biology, Swedish University of Agricultural Sciences, Alnarp 230 53, Sweden

#Corresponding Author: Amit Roy, phone: (+420) 224383565, Email: Roy@fld.czu.cz;

Supplementary Table Legends

Supplementary Table 1:

ANOSIM and MRPP analyses representing the extent of variation among the gut bacteriome of bark beetles. Positive R values in ANOSIM analysis represents significant differences between the bacterial communities in bark beetle gut. The less observe-delta value in MRPP analysis indicates low variation in gut bacterial communities within the biological replicates of each beetle while higher expected-delta values denote larger differences between the bark beetles. A positive A-value suggests that variation among beetles is larger than variation within groups. Significance value < 0.05 indicates significant differences between the bark beetle gut bacteriome.

Supplementary Table 2:

ADONIS analysis based on Bray-Curtis method to estimate the significant difference between different bark beetles. Df denotes degree of freedom, SS represents sums of squares of deviations, MS stands for SS/Df. F. Model represents F-test value. The R2 value illustrates the ratio of grouping variance and total variance. Values in parentheses stands for Residual Error. The p-value determines the significant variation between the bark beetles.

Supplementary Table 3:

Analysis of molecular variance (AMOVA) determining differences of microbial community structure among bark beetles. SS stands for sums of squares of deviations. df denotes degree of freedom. MS represents SS/Df. Fs is the F-test value. Values in parentheses stands for Residual Error. The p-value determines the significant variation between the bark beetles.

Supplementary Figure Legends

Supplementary Figure 1:

Rarefaction Curves. Different colors and symbols denote different samples.

Supplementary Figure 2:

(A) The evolutionary tree representing the top 100 genera. Different colors of the branches indicate different phyla. Relative abundance of each genus in each bark beetle is displayed outside the circle with different colors denoting different beetles. (B) Taxonomy tree illustrating the top 10 genera in high relative abundance. Sectors with different colors represent different bark beetles and the size of the sector indicates the relative abundance. The first number below the taxonomic name denotes the percentage in the whole taxon, while the second number represents the percentage in the selected taxon.

Supplementary Figure 3:

Ternary plot representing the top 10 highly abundant bacterial species among (A) IT, ID and PP (B) SX, IC and IAC (C) IT, IC and SX (D) IC, ID and SX (E) PP, IC and SX. The three vertexes represent three bark beetles. The circles represent dominant species and the circle size indicates the relative abundance. The circles close to the bark beetles indicates higher abundance of that species in the particular beetle.

Supplementary Figure 4:

Box plot representing the β- diversity indices between bark beetles. (A) Boxplots based on Weighted Unifrac distance (B) Boxplot based on Unweighted Unifrac distance. Wilcoxon signed-rank test is performed for the analysis of significance of difference between groups. The same alphabets denote no significant differences.

Supplementary Figure 5:

Beta diversity analysis. (A) Weighted and Unweighted Unifrac distance matrices represent pairwise dissimilarity coefficient between bark beetles where Weighted Unifrac distance is displayed above, and Unweighted Unifrac distance denoted below. (B) Unweighted pair group method with arithmetic mean (UPGMA) tree cluster based on Unweighted Unifrac distance describes the similarity in gut bacterial communities among the bark beetles collected from R- site (IT, ID, IC, and SX). The UPGMA tree shown along with the relative abundance of bacterial communities at the phylum level. (C) Unweighted pair group method with arithmetic mean (UPGMA) tree cluster based on Weighted Unifrac distance representing the similarity in gut bacterial communities among the bark beetles feeding on spruce (PP, IT and ID). The UPGMA tree is shown with the relative abundance of bacterial communities at phylum level. [*Ips typographus* (IT), *Ips duplicatus* (ID), *Ips sexdentatus* (SX), *Ips acuminatus* (IAC), *Ips cembrae* (IC) and *Polygraphus poligraphus* (PP)].

Supplementary Figure 6:

T-test analysis illustrating significant bacterial species variation between spruce feeding bark beetle collected from R site (IT and ID). The left panel represents the mean abundance value of each bacterial species showing significant variation between IT and ID. The right panel shows the confidential interval of between group variation. The left-most part of each circle stands for the lower limit of 95% confidential interval, while the right-most part denotes the upper limit. The center of the circle stands for the difference of the mean value. The color of the circle denotes the bark beetle with higher mean value. The p-value of the significance test represents the variation between the beetles.

Supplementary Figure 7:

LEfSe analysis representing (A) histogram of the LDA scores illustrating the presence of gut bacterial species (biomarker) whose abundance differs significantly among the six bark beetles. The length of each bin i.e. LDA score represents the effect size (the extent to which a biomarker can explain the differentiating phenotypes among groups) at LDA score cutoff threshold >4. (B) The cladogram representing the bacterial biomarkers among all the bark beetles. The circles radiating from inside to outside designates the taxonomic level from phylum to genus. Each circle represents a distinct taxon at corresponding taxonomic level. The size of each circle is proportional to the relative abundance of each taxon. Bacterial species (biomarkers) with significant differences are colored according to color of corresponding bark beetle whereas yellowish green circles resemble non-significant bacterial species. Red and green nodes denote the that these bacteria contribute highly in the group. Letters above the circles describes the bacterial biomarker.

Supplementary Figure 8:

Histogram of the LDA scores illustrating the presence of gut bacterial species (biomarker) whose abundance differs significantly among (A) the spruce feeding bark beetles (IT, ID and PP) (B) pine feeding bark beetles (IAC and SX). The length of each bin i.e. LDA score represents the effect size (the extent to which a biomarker can explain the differentiating phenotypes among groups) at LDA score cutoff threshold >4. The color of each bin complies with the bark beetles.

Supplementary Excel Legends

Supplementary Excel 1: Raw and assembled read counts with read length.

Supplementary Excel 2: QC statistics and qualified clean read counts.

Supplementary Excel 3: The OTU table representing the OTU abundance in six bark beetles (with biological replicates).

Supplementary Excel 4: Relative abundance of gut bacteriome (phylum level and genus level) present in six bark beetles.

Supplementary Excel 5: The shared and unique bacterial communities comparing spruce feeding *Ips* bark beetles (IT and ID).

Supplementary Excel 6: The common core and unique bacterial communities comparing pine feeding bark beetles (IAC and SX).

Supplementary Excel 7: The common core and unique bacterial communities comparing all five *Ips* beetles.

Supplementary Table 1: ANOSIM and MRPP analysis.

Group*	ANOSIM		MRPP			
	R-value	P-value	A	observed-delta	expected-delta	Significance
IAC-SX	0.8611	0.002	0.2465	0.6047	0.8025	0.003
IT-SX	0.237	0.02	0.08894	0.5816	0.6383	0.029
IT-IAC	0.8139	0.001	0.1771	0.7017	0.8528	0.003
ID-SX	0.5704	0.001	0.156	0.5869	0.6954	0.008
ID-IAC	0.5981	0.005	0.1262	0.7071	0.8092	0.008
ID-IT	0.4444	0.018	0.05708	0.684	0.7254	0.115
PP-SX	1	0.001	0.5294	0.3238	0.6881	0.003
PP-IAC	0.6083	0.007	0.3285	0.444	0.6612	0.01
PP-IT	1	0.002	0.4235	0.4209	0.7301	0.002
PP-ID	1	0.003	0.4188	0.4263	0.7334	0.003
IC-SX	0.4926	0.003	0.1472	0.4656	0.546	0.009
IC-IAC	0.7463	0.003	0.2616	0.5858	0.7933	0.006
IC-IT	0.3556	0.004	0.1347	0.5627	0.6503	0.006
IC-ID	0.5222	0.002	0.1402	0.5681	0.6607	0.007
IC-PP	1	0.002	0.5503	0.305	0.6782	0.002

* *Ips typographus* (IT), *Ips duplicatus* (ID), *Ips sexdentatus* (SX), *Ips acuminatus* (IAC), *Ips cembrae* (IC) and *Polygraphus poligraphus* (PP)

Supplementary Table 2: ADONIS analysis.

Group*	Df	SS	MS	F. Model	R2	p- value (>F)
IAC-ID	1(10)	1.0660(2.7183)	1.06603(0.27183)	3.9217	0.2817(0.7183)	0.003
IAC-SX	1(10)	1.7688(2.0899)	1.76881(0.20899)	8.4634	0.45839(0.54161)	0.001
IAC-PP	1(10)	1.6760(1.4872)	1.67601(0.14872)	11.27	0.52984(0.47016)	0.004
IAC-IC	1(10)	1.8243(1.9563)	1.82434(0.19563)	9.3253	0.48254(0.51746)	0.001
IAC-IT	1(10)	1.4608(2.8262)	1.46077(0.28262)	5.1687	0.34075(0.65925)	0.001
ID-SX	1(10)	1.0229(1.9861)	1.02288(0.19861)	5.1501	0.33994(0.66006)	0.004
ID-PP	1(10)	2.2448(1.3834)	2.24482(0.13834)	16.227	0.61871(0.38129)	0.002
ID-IC	1(10)	0.81658(1.85254)	0.81658(0.18525)	4.4079	0.30594(0.69406)	0.003
ID-IT	1(10)	0.5854(2.7224)	0.58540(0.27224)	2.1503	0.17697(0.82303)	0.072
SX-PP	1(10)	2.5723(0.7551)	2.57232(0.07551)	34.068	0.77308(0.22692)	0.004
SX-IC	1(10)	0.58777(1.22419)	0.58777(0.12242)	4.8013	0.32438(0.67562)	0.002
SX-IT	1(10)	0.53384(2.09407)	0.53384(0.20941)	2.5493	0.20314(0.79686)	0.043
PP-IC	1(10)	2.6248(0.6215)	2.62476(0.06215)	42.236	0.80856(0.19144)	0.001
PP-IT	1(10)	2.1813(1.4913)	2.18134(0.14913)	14.627	0.59394(0.40606)	0.001
IC-IT	1(10)	0.7217(1.9605)	0.72170(0.19605)	3.6813	0.26908(0.73092)	0.006

* *Ips typographus* (IT), *Ips duplicatus* (ID), *Ips sexdentatus* (SX), *Ips acuminatus* (IAC), *Ips cembrae* (IC) and *Polygraphus poligraphus* (PP)

Supplementary Table 3: AMOVA analysis.

Group*	SS	df	MS	Fs	p-value
IC-IT	0.203704(0.241047)	1(10)	0.203704(0.0241047)	8.45079	<0.001*
IT-PP	0.0406259(0.036644)	1(10)	0.0406259(0.0036644)	11.0866	0.003*
IAC-IT	0.354745(0.447015)	1(10)	0.354745(0.0447015)	7.93587	0.014
IC-PP	0.275915(0.216024)	1(10)	0.275915(0.0216024)	12.7724	<0.001*
ID-PP	0.0848206(0.126081)	1(10)	0.0848206(0.0126081)	6.72748	<0.001*
IAC-PP	0.392635(0.421992)	1(10)	0.392635(0.0421992)	9.30431	0.007
IAC-SX	0.359865(0.426533)	1(10)	0.359865(0.0426533)	8.43699	0.008
IT-SX	0.00901791(0.0411847)	1(10)	0.00901791(0.00411847)	2.18962	0.083
IAC-IC	0.285237(0.626395)	1(10)	0.285237(0.0626395)	4.55362	0.009
ID-SX	0.0505416(0.130621)	1(10)	0.0505416(0.0130621)	3.86932	0.004
IAC-ID	0.192886(0.536452)	1(10)	0.192886(0.0536452)	3.59558	0.043
IC-ID	0.152913(0.330484)	1(10)	0.152913(0.0330484)	4.62695	0.008
PP-SX	0.0396845(0.0161617)	1(10)	0.0396845(0.00161617)	24.5546	0.002*
ID-IT	0.0428635(0.151104)	1(10)	0.0428635(0.0151104)	2.8367	0.013
IC-SX	0.194172(0.220565)	1(10)	0.194172(0.0220565)	8.80337	0.001*
IAC-IC-ID-IT-PP-SX	0.893209(0.793661)	5(30)	0.178642(0.0264554)	6.75257	<0.001*

* *Ips typographus* (IT), *Ips duplicatus* (ID), *Ips sexdentatus* (SX), *Ips acuminatus* (IAC), *Ips cembrae* (IC) and *Polygraphus poligraphus* (PP)

Supple Figure 1_ Rarefraction curves

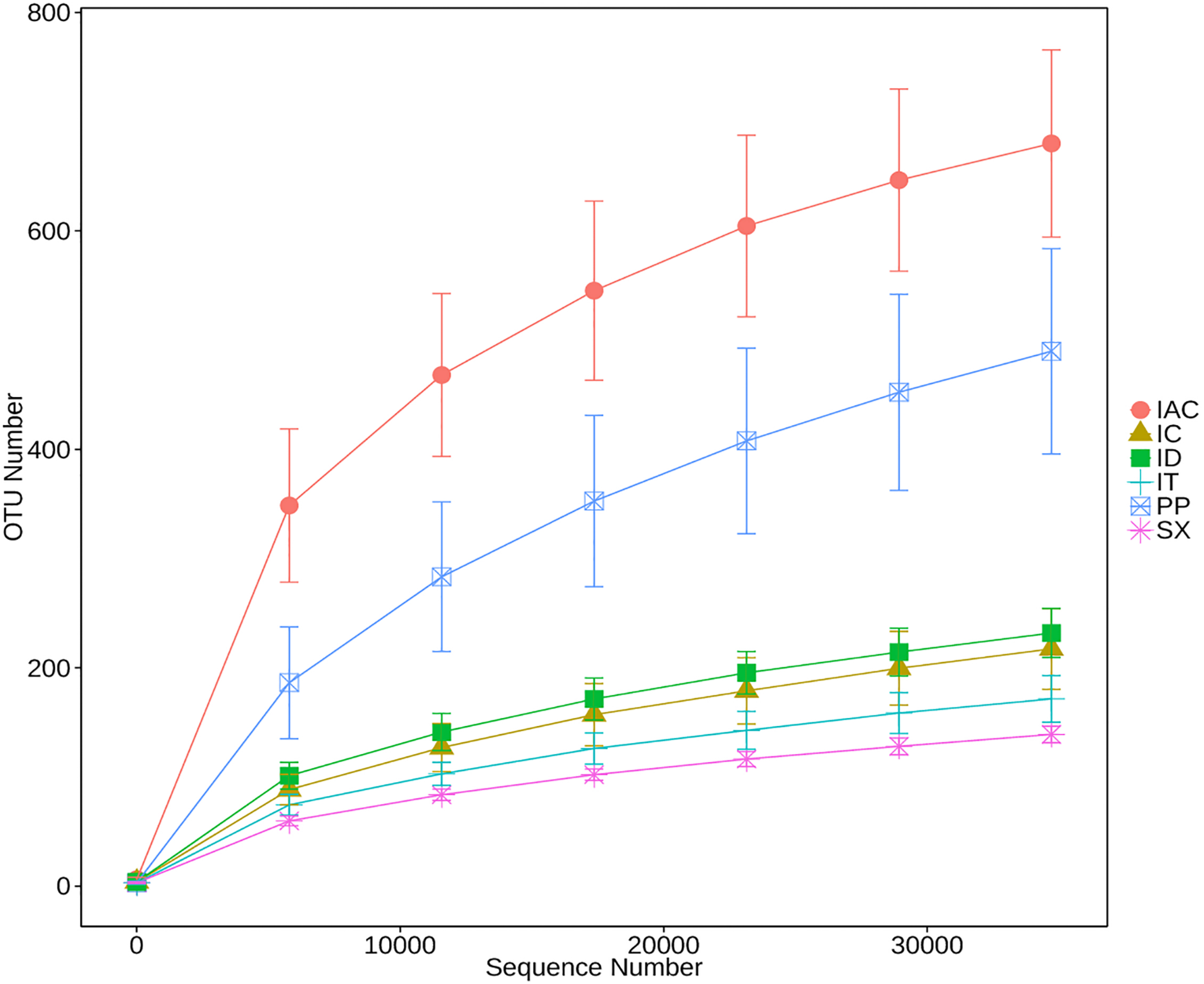

Supple Figure 2 _Evolutionary taxonomy tree

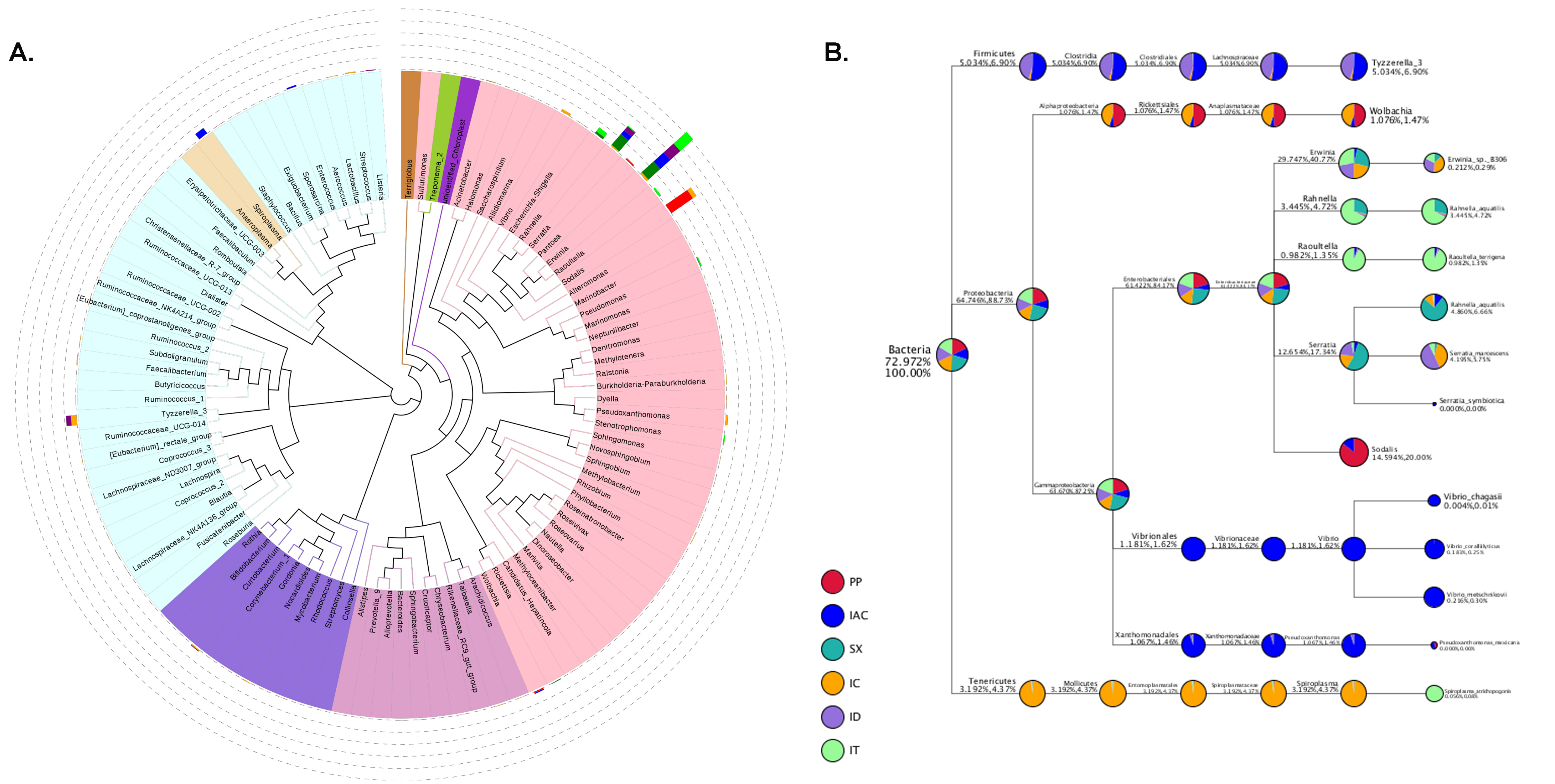

Supple Figure 3 _ Ternary plot

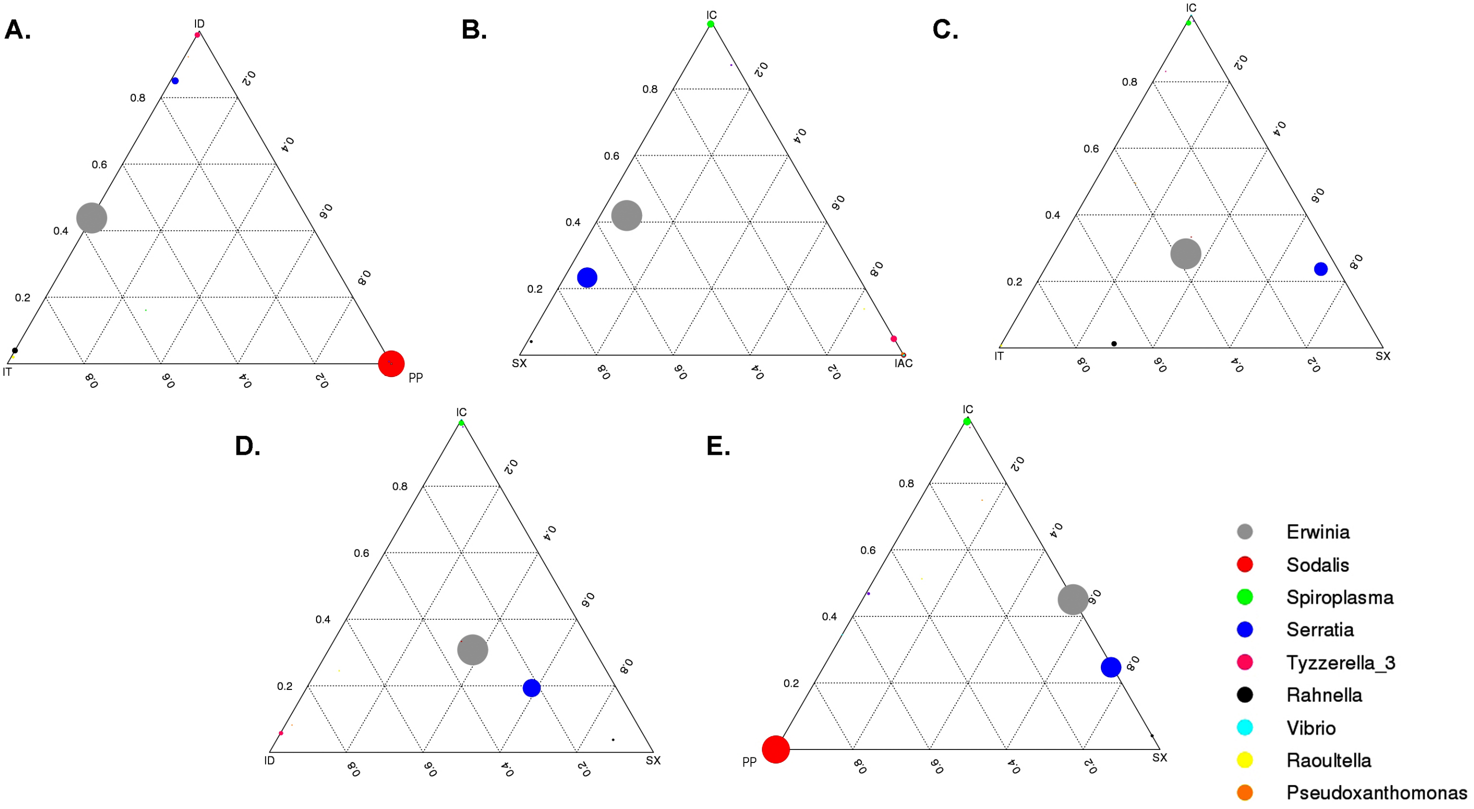

ternaryplot

Supple Figure 4 _Box plot Unifrac

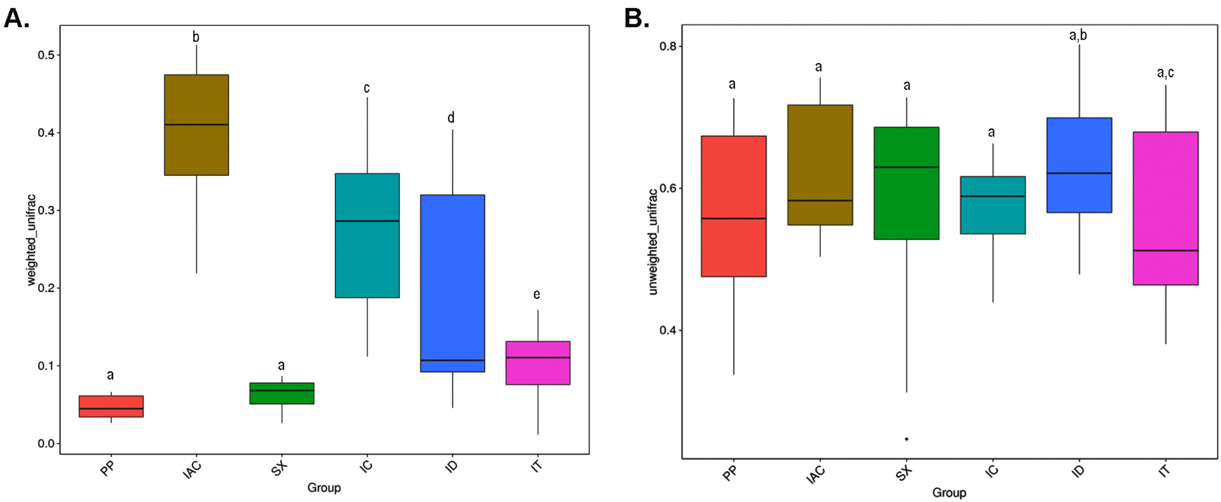

Supplementary fig: 5 (beta- diversity)

A.

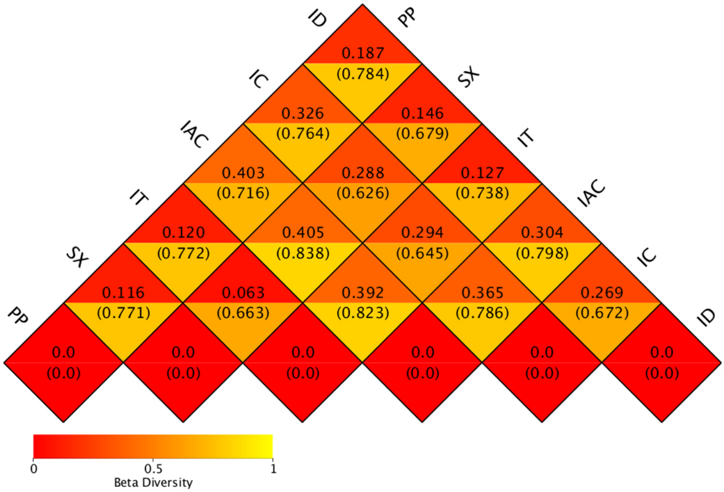

B.

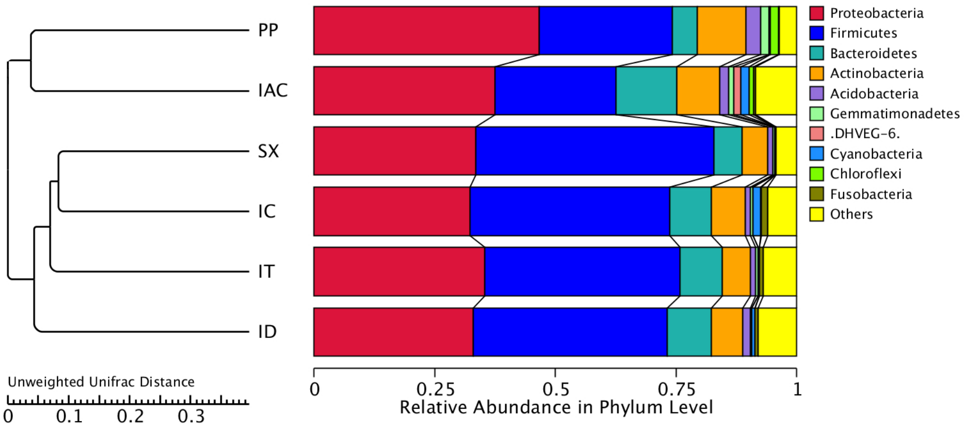

C.

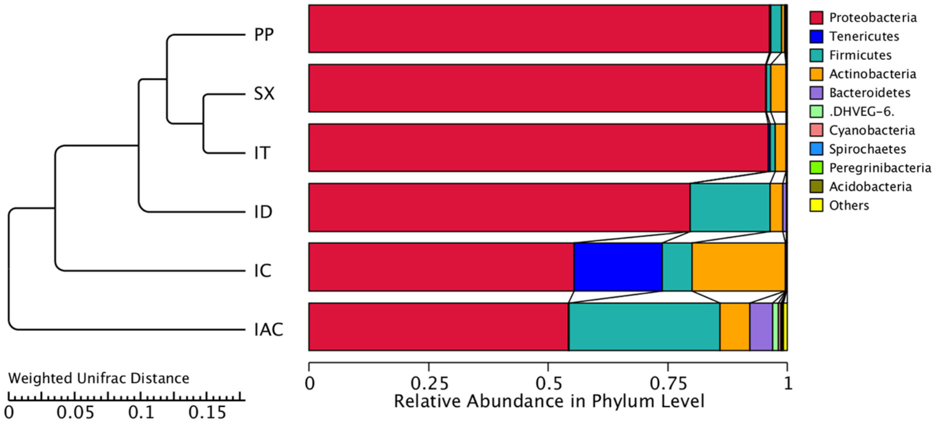

Supple Figure 6_ IT vs ID - T-test

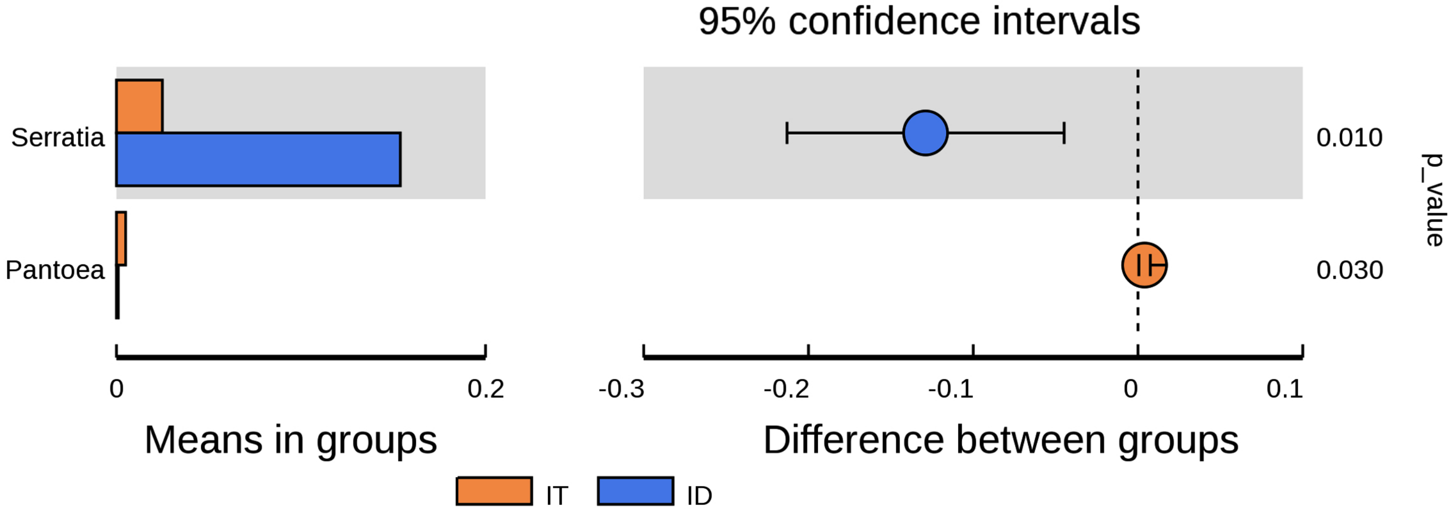

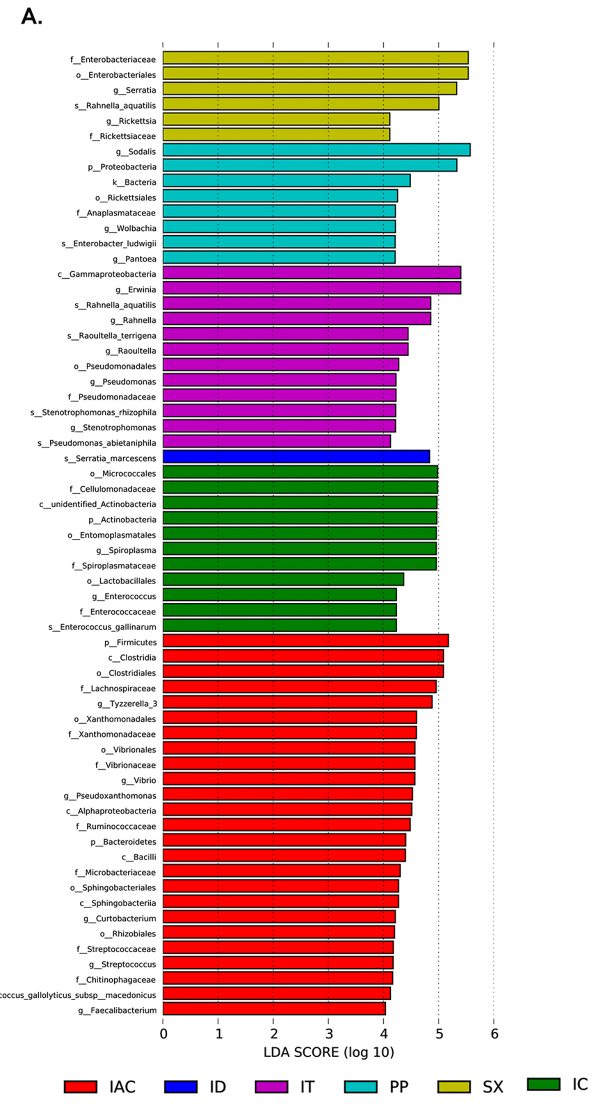

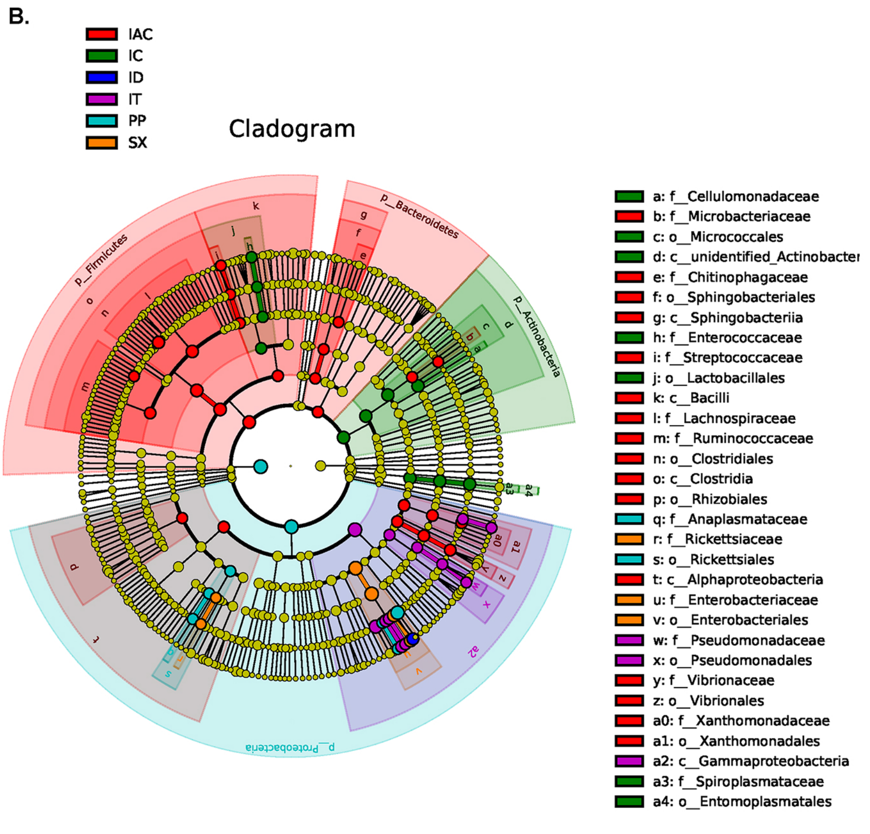

Supple Figure 7 _LefSe_all_beetles

Supple Figure 8 _LDA_Spruce_Pine_beetles

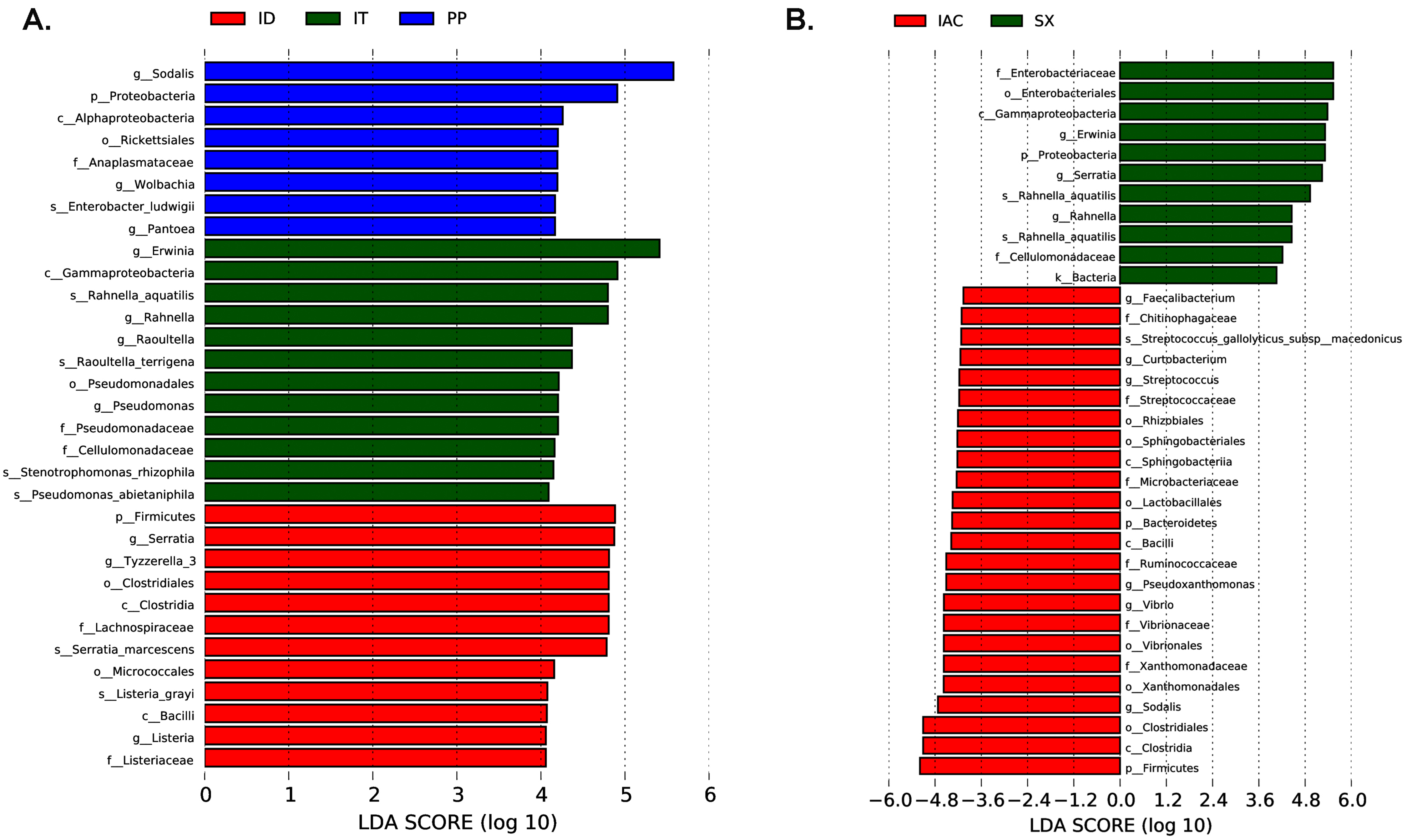

Supplement: Supplementary file 1 — Supplementary Information 1. [file 41598_2020_75203_MOESM1_ESM.pdf]
